# Single-cell RNA sequencing reveals aberrant airway epithelial–immune cell cross-talk in pulmonary fibrosis

**DOI:** 10.1183/23120541.01273-2025

**Published:** 2026-08-03

**Authors:** Richard J. Hewitt, Probir Chakravarty, Jimena Perez-Lloret, Martin Banchero, Marijn Berg, Maarten van den Berge, William J. Traves, Laura L. Yates, Simone A. Walker, David C.A. Gaboriau, Alexandra Rice, Andrew G. Nicholson, Anand Devaraj, Samuel V. Kemp, Philip L. Molyneaux, Franz Puttur, Adam J. Byrne, Toby M. Maher, Martijn C. Nawijn, Anne O'Garra, Clare M. Lloyd

**Affiliations:** 1 National Heart and Lung Institute, Imperial College London, London, UK; 2 Royal Brompton and Harefield Hospitals, Guy's and St Thomas’ NHS Foundation Trust, London, UK; 3 The Francis Crick Institute, Bioinformatics & Biostatistics STP, London, UK; 4 The Francis Crick Institute, Advanced Sequencing Facility STP, London, UK; 5 Department of Pathology & Medical Biology, University of Groningen, University Medical Center Groningen, GRIAC Research Institute, Groningen, the Netherlands; 6 Facility for Imaging by Light Microscopy, Imperial College London, London, UK; 7 The Francis Crick Institute, Laboratory of Immunoregulation & Infection, London, UK

## Abstract

**Background:**

Epithelial–immune cell interactions are crucial in the regulation of pulmonary immune responses. Emerging evidence suggests that cell populations lining the airways may play a pivotal role in the pathogenesis of idiopathic pulmonary fibrosis (IPF), a disease characterised by progressive scarring of the lung parenchyma. We profiled the cellular landscape of the airway mucosal niche in incident cases of IPF to understand early-stage events contributing to disease development.

**Methods:**

Single-cell RNA-sequencing was used to explore cellular heterogeneity in proximal airway brushings from seven healthy controls and nine patients with newly diagnosed IPF. In-depth bioinformatics analysis was used to interrogate changes in cell populations and cell–cell communication in IPF patients compared to controls.

**Results:**

We show a relative increase in the abundance of airway macrophage subsets in IPF compared to healthy controls, and disease-specific changes in their transcriptional profile. Increased frequency of airway macrophages and proliferating macrophages was associated with more extensive disease at baseline quantified by the composite physiological index and radiological severity of traction bronchiectasis. Monocyte-derived macrophages were significantly enriched at baseline in IPF patients who had disease progression at 12 months. Using CellChat we exposed differences in cell–cell communication between airway epithelial cells, airway macrophages and T-cells in IPF. We identified dysregulation in signalling pathways such as SEMA3, ANXA1 and DESMOSOME, which modulate airway epithelial–macrophage interactions, potentially driving disease pathology.

**Conclusions:**

Airway epithelial cells and macrophages may play a key role in orchestrating the early immunopathology of IPF, and these data support further exploration of novel, airway-focused therapeutic targets in IPF.

## Shareable abstract

**Single-cell RNA-seq reveals that cell–cell interactions between airway epithelial cells and airway macrophages play a key role in driving early immunopathology in newly diagnosed cases of idiopathic pulmonary fibrosis** https://bit.ly/4iaVbRn

## Introduction

Specialised epithelial and immune cells lining the human airways play a vital role in lung host defence [1]. This diverse community of cells must sense and respond to inhaled pollutants, toxins and microbes to prevent cellular injury in the distal airspace [2]. Idiopathic pulmonary fibrosis (IPF) is a progressive and incurable scarring lung disease of older adults, postulated to be caused by a dysregulated repair response to repetitive microinjury of the alveolar epithelium [3]. Irreversible fibrosis leads to alveolar destruction, architectural remodelling, impaired gas exchange and respiratory failure. The strongest genetic risk factor for IPF is the promoter variant rs35705950 in *MUC5B*, a gene encoding a mucin glycoprotein secreted by airway epithelial cells, which plays a critical role in mucosal immunity [4, 5]. Emerging evidence suggests that structural and cellular changes in the airways may precede fibrosis in the lung interstitium [6]. Transcriptomic changes have been reported in macroscopically normal-appearing lung tissue [7] and the nasal epithelium [8] in IPF, supporting the concept that the disease is not only limited to areas of lung fibrosis.

The cellular landscape of the distal fibrotic lung has been unveiled through single-cell RNA sequencing (scRNA-seq) studies using lung tissue explants from donors with end-stage IPF [9–15]. These studies demonstrate a shift in cellular composition in the distal lung in IPF, with a reduction in alveolar epithelial cells and an increase in airway epithelial cell types [9, 11, 12, 14]. Unique populations of aberrant basaloid epithelial cells [11, 12], alveolar macrophages [10, 11, 14] and fibroblasts [12, 15] in tissue explants have been implicated in the pathobiology of advanced IPF. Spatial transcriptomics has unveiled the cellular composition and molecular changes within distinct pathological niches in advanced fibrosis [16]. However, early-stage disease in the airways has not been studied at the single-cell level and will provide insight into transcriptomic changes and cell–cell interactions that contribute to disease initiation and progression.

Single-cell transcriptomics has been used to study purified airway epithelial cells from airway brushings in IPF compared to interstitial lung disease controls [17]. However, a comprehensive single-cell analysis of the proximal airway mucosal niche in IPF has not been reported. Here, we used scRNA-seq to compare epithelial and immune cell populations in nonmanipulated brushings from the proximal airway wall in newly diagnosed, treatment-naïve IPF patients compared to healthy controls. We exposed differences in immune cell-type abundance with differential gene expression and discovered novel interactions between multiciliated epithelial cells and airway macrophages in IPF. Increased abundance of airway and proliferating macrophages correlated with disease extent and thus may provide avenues for exploring further airway-focused therapeutic interventions.

## Methods

Further details are provided in the supplementary material.

### Study population

Heathy subjects and IPF patients were prospectively recruited between 2017 and 2020 at the Royal Brompton Hospital (London, UK). Healthy control subjects included ex-smokers and nonsmokers with no history of lung disease and normal lung function (forced expiratory volume in 1 s (FEV_1_) >80% of the predicted value for age and height, FEV_1_/forced vital capacity (FVC) ratio >70%). Patients with IPF diagnosed after multidisciplinary discussion according to international guidelines [18], with FVC ≥50% of the predicted value, and a diffusion capacity of the lung for carbon monoxide ≥30% pred value, were included. Subjects with a history of respiratory tract infection, antibiotic use or acute exacerbation within the past 3 months were excluded. Subjects taking antifibrotic therapy, concomitant steroids, other immunomodulatory treatments or supplementary oxygen were excluded. All subjects underwent fibreoptic bronchoscopy with proximal airway brushings in accordance with a standard operating procedure [19]. Ethical approval was granted by the research ethics committee (NRES: 15/SC/0101 and 15/LO/1399) and all subjects provided written informed consent.

### Single-cell RNA-seq

A single-cell suspension was generated from airway brushings using an established protocol [20]. Single-cell capture and RNA-seq was performed using the 10X Genomics Chromium platform v3.1. Raw reads were processed using the 10X Cell Ranger v3.0.2 and subsequent analyses performed in R v3.6.0 using Seurat v3.0 [21] (further details are provided in the supplementary material). Cluster specific markers were identified using the “FindMarkers” function using default settings (min.pct=0.25 and logfc.threshold=0.25), which uses the Wilcoxon rank sum test to compare each cell belonging to one cluster *versus* all other cells. The Human Lung Cell Atlas reference dataset [22] was used for cluster annotation. Common anchors between our dataset and that of the Human Lung Cell Atlas were identified and data projected using the first 30 components of the principal component analysis reduction space. Human Lung Cell Atlas level 5 annotations were used for label transfer with the “MapQuery” function of Seurat. Cell type annotation was further refined by comparing cluster marker gene expression pattern with canonical markers and cell signatures reported in published cell atlases [20, 23, 24]. Cell abundance testing was performed using three methods outlined in the supplementary material. Differentially expressed genes between IPF and healthy per cluster were identified using the function “findallmarkers” within the Seurat package, using a Benjamini–Hochberg (false discovery rate (FDR)) adjusted p-value of <0.05 and absolute(log_2_FC) of 0.25. Using pseudobulk data per patient we have drawn heatmaps representing selected differentially expressed genes per cluster. Each row within the heatmap is scaled and the deviation of expression per patient from the median is colour coded. To describe the gene changes per cell type, differentially expressed genes were used for an overenrichment analysis for Gene Ontology biological processes, using a hypergeometric test, with the ClusterProfiler package [25]. To infer cell–cell communication in the airway we used CellChat (version 1.1.3) [26]. Additional detail on the scRNA-seq data analysis is provided in the supplementary material.

### Lung histology

Archived formalin-fixed paraffin-embedded (FFPE) lung tissue sections of proximal airways were obtained with ethical approval (NRES: 15/SC/0569). All specimens were reviewed by two consultant histopathologists. Normal parenchymal lung tissue was obtained during localised lung cancer resection. IPF tissue samples were procured from explant lung tissue or surgical lung biopsies. Detailed methods on imaging mass cytometry and immunofluorescence microscopy are provided in the supplementary material.

## Results

### The cellular landscape of the airway niche in IPF and healthy controls

**FIGURE 1:**
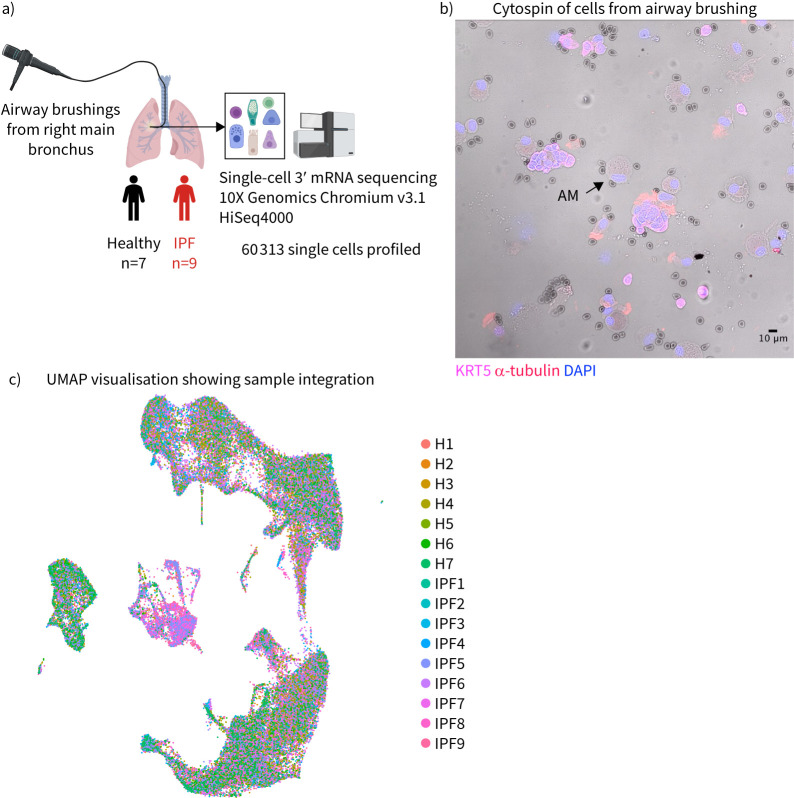
Single-cell analysis of airway brushings from healthy controls and patients with idiopathic pulmonary fibrosis (IPF). a) Airway brushings were collected *via* bronchoscopy from nine newly diagnosed IPF patients and seven healthy controls and single-cell 3′ mRNA sequencing performed using 10X Genomics Chromium v3.1. b) Cytospin immunofluorescence microscopy of airway brushing showing heterogeneity in cell type; keratin (KRT)5^+^ basal cells, α-tubulin^+^ multiciliated cells, airway macrophages (AM) indicated by arrow, 4′,6-diamidino-2-phenylindole (DAPI). Scale bar=1 μm. c) Uniform manifold approximation and projection (UMAP) representation of the whole dataset with cells coloured according to subject.

We analysed bronchial brushings collected from the right main bronchus of seven healthy controls and nine newly diagnosed cases of IPF (figure 1a). Subjects with IPF were sampled early in the disease course, were all treatment-naïve and did not require long-term oxygen therapy (table 1 and supplementary table S1). Disease extent was determined through calculation of composite physiological index (CPI) derived from lung function values [27], and scoring of high-resolution computed tomography (HRCT) scans as described previously [28] (table 1 and supplementary table S1). One IPF patient had honeycombing on HRCT scan review (supplementary table S1). Progressive disease (≥10% decline in FVC or death at 12 months) was identified in four out of nine IPF patients (supplementary table S2). At 3-year follow-up, six out of nine patients with IPF had died of respiratory failure (supplementary table S2).

**TABLE 1:** Baseline characteristics of idiopathic pulmonary fibrosis (IPF) patients and healthy controls

	Healthy control	IPF
**Participants, n**	7	9
**Age, years**	45.7±9.4	69.2±10.1
**Male/female**	3/4	9/0
**Smoking status**		
Ex-smoker	4	3
Never-smoker	3	6
**FEV_1_ % predicted**	92.6±10.2	87.0±18.7
**FVC % predicted**	91.3±8.3	77.2±15.0
**FEV_1_/FVC %**	82.1±3.9	85.8±4.8
***D*_LCO_ % predicted**	NA	49.2±10.8
**CPI**	NA	47.7±8.3
**Fibrosis extent, %**	NA	27.2±11.8
**Traction bronchiectasis severity score**	NA	6.2±3.2

FEV_1_: forced expiratory volume in 1 s; FVC: forced vital capacity; *D*_LCO_: diffusing capacity of the lung for carbon monoxide; CPI: composite physiological index; NA: not applicable.

We interrogated the airway mucosal niche using single-cell RNA-seq to characterise cellular diversity and relate cell abundance to the extent of disease pathology. We profiled 60 313 cells including 22 487 from healthy controls and 37 826 from patients with IPF. Cytospin immunostaining confirmed that airway brushings captured both epithelial and immune cell populations from the airway mucosal surface including α-tubulin^+^ ciliated cells, KRT5^+^ basal cells and airway macrophages (figure 1b). Dimensionality reduction with uniform manifold approximation and projection (UMAP) visualisation showed good sample integration with representation of clusters across donors with no discernible batch effect (figure 1c).

**FIGURE 2:**
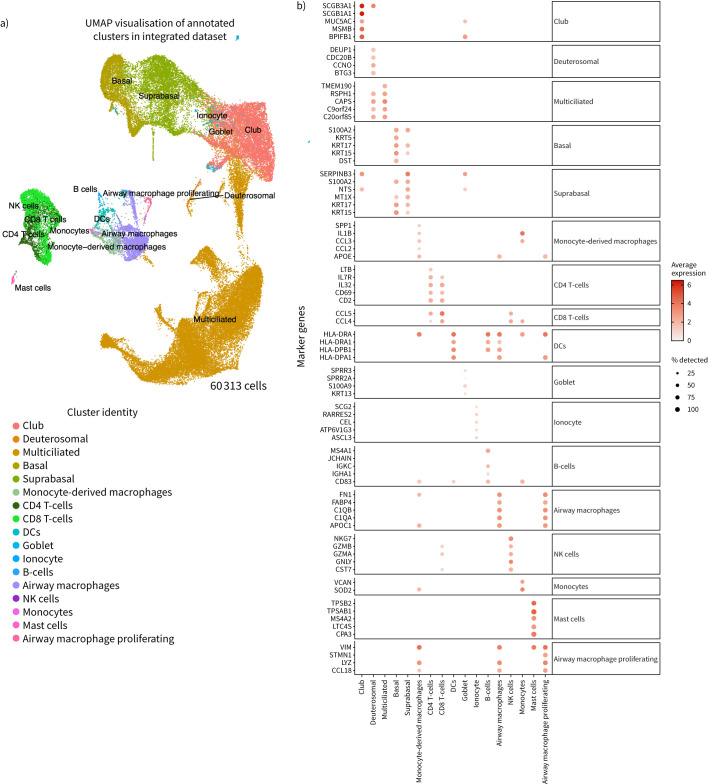
Distinct airway epithelial cell and immune cell populations revealed by single-cell RNA-sequencing. a) Uniform manifold approximation and projection (UMAP) representation of 60 313 cells from idiopathic pulmonary fibrosis (IPF) patients and healthy controls, clustered based on gene expression. Colour specifies assignment of cells to one of 17 clusters with labels transferred from level 5 annotation of the Human Lung Cell Atlas [22]. b) Dot plot comparing expression of top marker genes across cell clusters, where x-axis denotes cluster and y-axis denotes marker genes. Dot size is proportional to the percentage of cells expressing the gene in each cluster. Colour intensity is proportional to averaged scaled log-normalised expression within a cluster. NK: natural killer; DC: dendritic cell.

Unbiased clustering revealed seven epithelial cell clusters and 10 immune cell clusters, which were labelled by annotation transferred from the Human Lung Cell Atlas and refined manually based on marker gene expression (figure 2a and 2b). Multiciliated cells comprised the largest epithelial population with high expression of *RSPH1*, which plays an important role in motility of cilia, and *C9orf24/SPMIP6*, which is involved in epithelial differentiation and ciliogenesis. Club cells were distinguished by high expression of members of the secretoglobulin family (*SCGB1A1*, *SCGB3A1*). Rare epithelial cell types were identifiable, including ionocytes expressing *ASCL3* and *CFTR* [29], and deuterosomal cells [23, 30, 31] expressing genes required for centriole amplification and ciliogenesis (*CDC20B*, *CCNO*).

In the healthy airway, CD8 T-cells expressing markers of tissue-residency, including *CD69* and *ITGA1* [32], were the most abundant immune cell type (figure 2a and b). In contrast, airway macrophages with a transcriptional profile which overlapped with resident airspace populations (described previously [33]) predominated in IPF. Airway macrophages expressed genes associated with homeostatic functions such as lipid transport and metabolism (*APOC1*, *FABP4*, *APOE*), iron handling (*FTL*)*,* phagocytosis (*MARCO*, *MRC1*, *MSR1*) and antigen presentation (*HLA-DPB1*) [34, 35]. We discovered two further airway macrophage subsets with unique gene expression profiles capturing distinct cell states akin to those reported previously [35]. Monocyte-derived macrophages were characterised by high expression of *SPP1,* an established profibrotic marker [35, 36], inflammatory cytokines (*IL1B*, *CCL3*), monocyte chemoattractant (*CCL2*) and members of the cysteine cathepsin family (*CTSB*, *CTSL*, *CTSZ*)*,* which are involved in intracellular protein turnover and extracellular matrix remodelling. Proliferating airway macrophages expressed cell cycle associated genes (*STMN1*, *TOP2A*, *MKI67*) and were found almost exclusively in the IPF airway*.* Natural killer (NK) and B-cells were present at low frequency.

### Increased abundance of airway macrophage subsets in IPF is associated with increased disease severity

**FIGURE 3:**
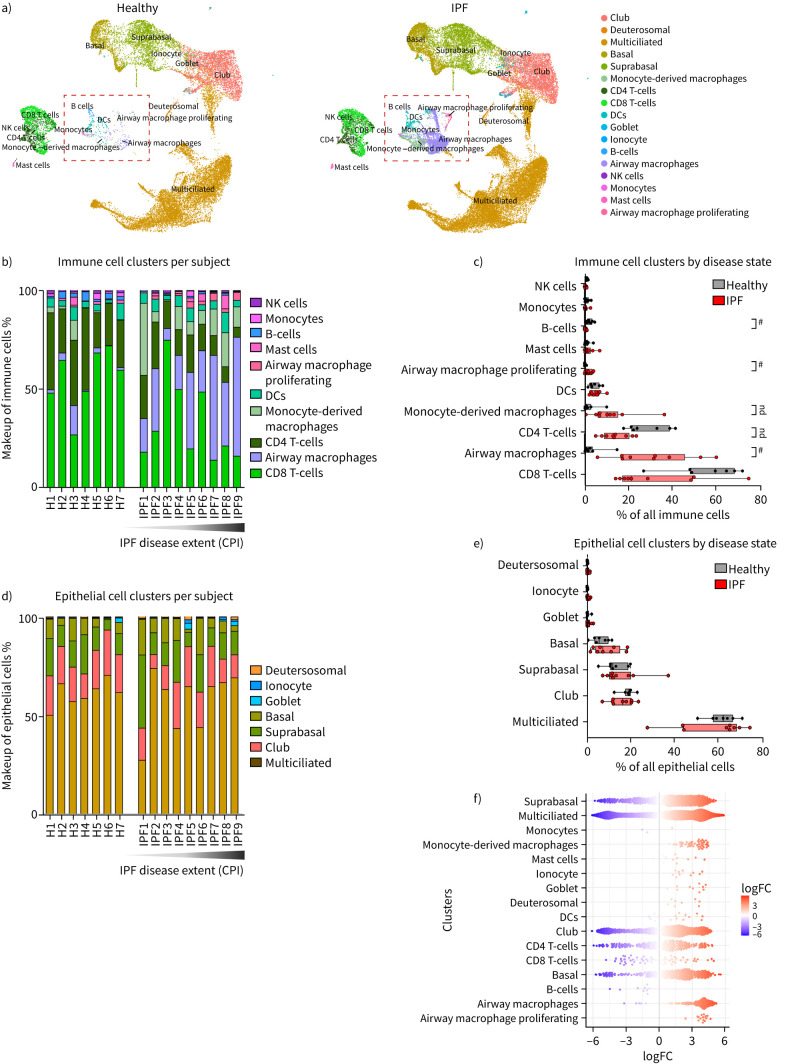
Relative increase in macrophage populations in the airways of patients with idiopathic pulmonary fibrosis (IPF). a) Uniform manifold approximation and projection (UMAP) representation of airway brushings split according to disease state with 22 487 cells from healthy controls (left panel) and 37 826 from patients with IPF (right panel), colour specifies cluster assignment. b) Bar chart showing relative percentage of cells within each immune cell cluster by individual subject. IPF patients listed in order of increasing disease extent (left to right) as indicated by composite physiological index (CPI). c) Boxplot showing relative percentage of cells within each immune cell cluster per individual subject comparing IPF (n=9) with healthy control (n=7). d) Bar chart showing relative percentage of cells within each epithelial cell cluster by individual subject. IPF patients listed in order of increasing disease extent (left to right) as indicated by CPI. e) Boxplot showing relative percentage of cells within each epithelial cell cluster per individual subject comparing IPF (n=9) with healthy control (n=7). For the boxplots, Mann–Whitney test and the Benjamini–Hochberg false discovery rate (FDR) method of multiple comparison correction is used with a desired FDR (Q) 1%. Those with a q-value (FDR-adjusted p-value) <0.05 are indicated on the figure: ^#^: a “discovery” q-value <0.01, nd: “not a discovery” q ≥0.01. f) Dot plots showing changes in neighbourhood abundance in airway subpopulations in IPF *versus* healthy controls using miloR. LogFC, coloured by spatial FDR <0.1. Cell clusters with significantly increased abundance in IPF are shown in red, while those with significantly decreased abundance are shown in blue.

The immune cell compartment was expanded in IPF airway brushings compared to healthy controls (supplementary figure S1). This could be appreciated when comparing UMAPs per individual IPF patient and healthy controls (supplementary figure S2). UMAP embedding of single cells from IPF and healthy airway brushings demonstrated an increase in the abundance of macrophage subsets in IPF compared to healthy controls (figure 3a). This was most significant in the mature, tissue-resident airway macrophage subset, but also evident with proliferating airway macrophages and monocyte-derived macrophages (figure 3b and c, supplementary table S3). Proliferating airway macrophages were present at low frequency in the majority of IPF patients (eight out of nine patients), but completely absent in all but one healthy control. There was a relative decrease in the abundance of B-cells in IPF compared to healthy controls (figure 3b and c, supplementary table S3). There were no significant differences in epithelial cell type abundance between IPF patients and healthy controls (figure 3d and e, supplementary table S3). Milo [37], a computational method which tests differential abundance of cells using k-nearest neighbour graphs, was used to confirm this increase in cell abundance in airway macrophages, proliferating airway macrophages and monocyte-derived macrophages in IPF relative to healthy controls (figure 3f, supplementary table S4). In addition, we identified an expansion of profibrotic *SPP1*^hi^ macrophages [14], in airway brushings from IPF relative to healthy controls (supplementary figure S3).

Re-analysing our data using only male samples to ensure there was no sex-bias, we found reproducible perturbations in airway macrophage subsets (supplementary figure S4). There was no correlation between age and cell type proportions in healthy controls, but in IPF patients, advancing age was associated with a decrease in airway macrophage abundance (supplementary figure S5).

**FIGURE 4:**
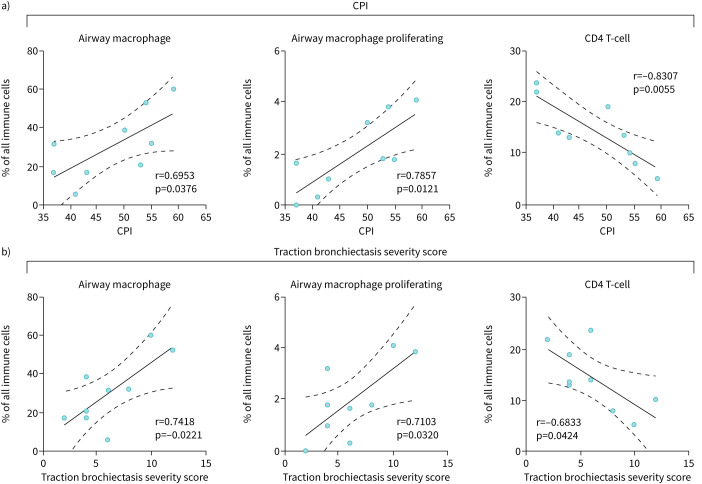
Airway macrophage subsets and CD4 T-cells correlate with idiopathic pulmonary fibrosis (IPF) disease severity. a) Pearson correlation analysis between composite physiological index (CPI) and relative percentage airway macrophages, proliferating airway macrophages and CD4 T-cells in IPF airway brushings. b) Pearson correlation analysis between traction bronchiectasis severity score and relative percentage airway macrophages, proliferating airway macrophages and CD4 T-cells in IPF airway brushings. Correlation coefficient, r, and two-tailed p-value noted on plot. Simple linear regression with best-fit line and 95% confidence bands shown.

Next, we analysed the relationship between cell type abundance and baseline disease severity. A higher abundance of airway macrophages and proliferating airway macrophages in IPF correlated with a higher CPI and computed tomography (CT) traction bronchiectasis severity score at baseline (figure 4). Conversely, a higher proportion of CD4 T-cells in IPF was associated with a lower CPI and CT traction bronchiectasis severity score (figure 4). There was a significant enrichment in monocyte-derived macrophages at baseline in IPF patients who progressed at 12 months compared to those who did not progress (supplementary table S3). There were no significant associations between baseline airway immune cell proportions and smoking status or survival at 3 years (supplementary table S3).

### Marked transcriptomic changes in airway macrophage subsets in IPF compared to healthy controls

**FIGURE 5:**
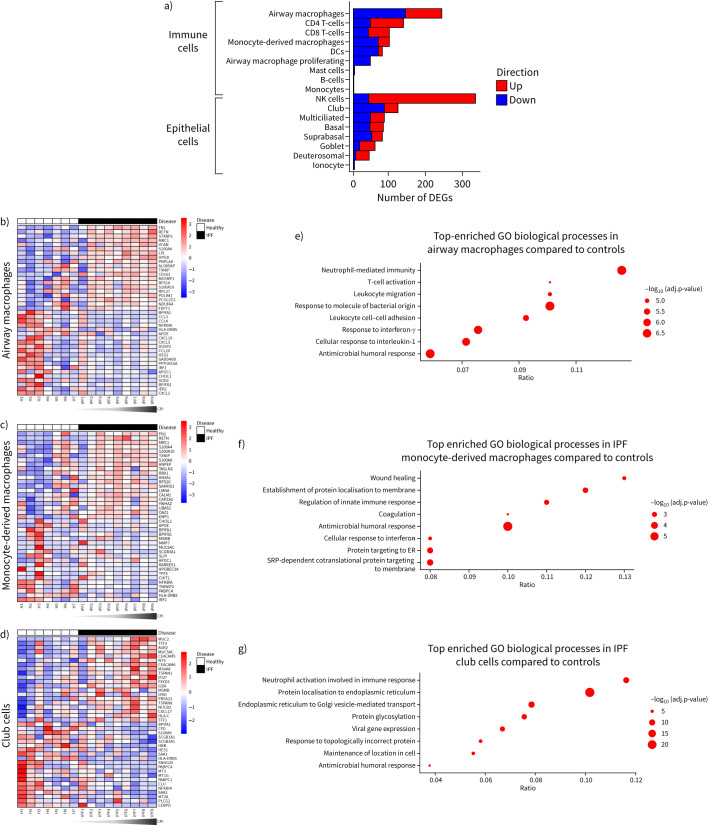
Marked transcriptomic changes in airway macrophages and club cells in idiopathic pulmonary fibrosis (IPF) compared to healthy controls. a) Number of differentially expressed genes (DEGs) comparing IPF with healthy controls for each cell type (log fold change (FC) cut-off of 0.25 and Benjamini–Hochberg (FDR) adjusted p-value <0.05). b–d) Heatmaps showing select top upregulated and downregulated genes in b) airway macrophages, c) monocyte-derived macrophages and d) club cells comparing IPF with healthy controls. The heatmap is coloured based on the z-score where we have calculated the deviation of normalised expression from the median value per gene. e–g) Dot plots showing enrichment of the top Gene Ontology (GO) biological processes using differentially expressed genes in e) airway macrophages, f) monocyte-derived macrophages and g) club cells comparing IPF with healthy controls. The size of the dot plot indicates the adjusted p-value of the enrichment, while the ratio indicates what percentage of the differential gene list that accounts for each GO biological process. DC: dendritic cell; NK: natural killer; CPI: composite physiological index; ER: endoplasmic reticulum; SRP: signal recognition particle.

After establishing cellular diversity within the airway mucosal microenvironment, we assessed the transcriptomic changes occurring in airway cell populations in IPF compared to healthy controls. The greatest number of differentially expressed genes (DEGs) were found in club cells (>300 genes) followed by airway macrophages (absolute log fold change >abs (0.25), Benjamani–Hochberg (FDR) adjusted p*-*value <0.05) (figure 5a). There was a low number of DEGs in proliferating macrophages because these cells were absent in all but one healthy control subject. To validate our analysis, we compared the expression profile of DEGs at an individual subject-level using a pseudobulk approach (figure 5b, supplementary table S5). IPF airway macrophages and monocyte-derived macrophages showed increased expression of *FN1* gene encoding fibronectin an adhesive glycoprotein involved in cell–matrix interactions [38], tissue remodelling [39] and elevated in IPF bronchoalveolar lavage (BAL) fluid [40] (figure 5b and c, supplementary table S5). Gene ontology (GO) biological processes associated with “neutrophil mediated immunity”, “T cell activation” and “leukocyte migration” were enriched in IPF airway macrophages compared to controls (figure 5e). “Wound healing” was one of the top enriched GO biological processes when looking at genes that are differentially regulated between monocyte-derived macrophages from IPF relative to healthy controls (figure 5f). In IPF club cells, there was a shift to a more goblet cell-like phenotype, with lower expression of typical club cell genes (*SCGB1A1*, *SCGB3A1*, *SAA1*) and greater goblet cell gene expression (*CEACAM5/6*, *MUC5AC*, *MUC2*) compared to controls (figure 5d, supplementary table S5). There was also enrichment in GO biological processes related to neutrophil activation and protein biosynthesis and transport in club cells from IPF patients’ brushings (figure 5g). Exclusion of female control subjects in the male-only differential gene expression analysis (supplementary figure S6) did not significantly alter these results. No significant DEGs were identified using a pseudobulk approach to compare clusters in IPF progressors *versus* IPF nonprogressors, possibly reflecting the numbers of patients under study (supplementary table S6). This was also the case for IPF ex-smokers *versus* IPF never-smokers (supplementary table S7). Collectively, these data indicate that airway macrophages and club cells play a pivotal role in processes linked to immune activation in the IPF airway.

### Increased cell–cell interactions in the airways of patients with IPF compared to healthy controls

**FIGURE 6:**
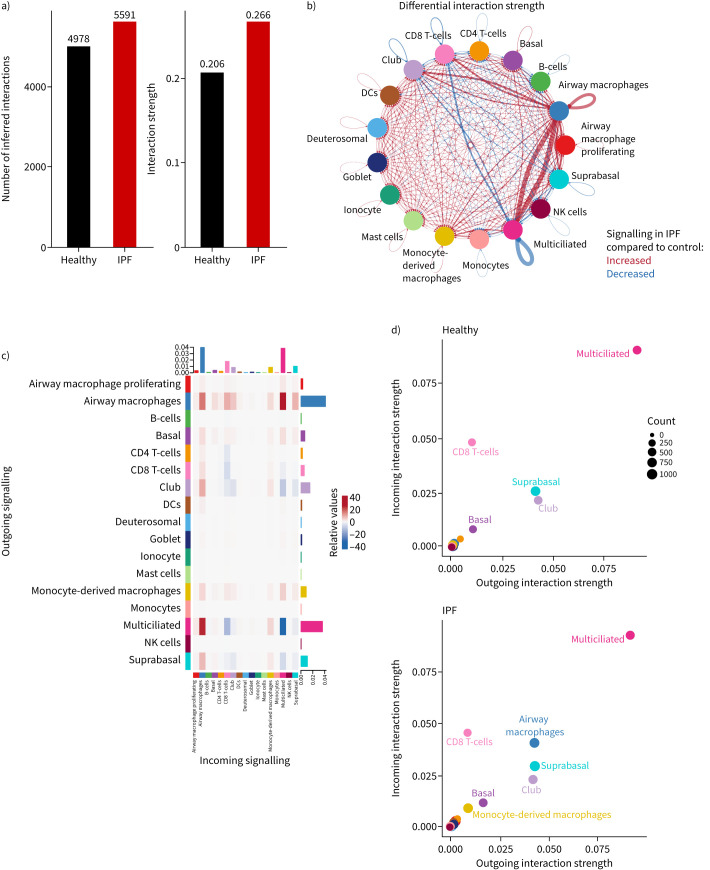
Increased cell–cell interactions in the airways of patients with idiopathic pulmonary fibrosis (IPF) compared to healthy controls. a) Bar charts showing the numbers and strength of cell-to-cell interactions inferred by CellChat within the grouped healthy and IPF datasets. b) Circle plot showing the difference in interaction strength between clusters in the IPF dataset relative to healthy dataset. The weight of edges represents the difference in interaction strength. The colour represents the direction of change relative to the healthy dataset. c) Heatmap showing the differential strength of interactions between clusters. Signals from sender clusters depicted in rows. Signals from receiver clusters depicted in columns. The bar plot at the top represents the sum of each column of absolute values displayed in the heatmap (incoming signalling). The right-hand bar plot represents the sum of each row of the absolute values (outgoing signalling). The bar height indicates the degree of change in interaction strength between IPF and healthy controls. Red: increased signalling strength in IPF compared to healthy control; blue: decreased signalling strength in IPF compared to healthy controls. d) Scatter plots showing the dominant sender (source) and receiver (target) cell populations between IPF and healthy controls. The x-axis shows total outgoing communication probability, and the y-axis shows total incoming communication probability associated with each cell group. Dot size is proportional to the number of inferred links (both outgoing and incoming) associated with each cell group. Dot colours indicate different cell groups.

Interactions between epithelial cells and immune cells within the airway are critical in the regulation of pulmonary immunity [2], but it is not known whether these influence the pathogenesis of IPF. To determine whether the relative increase in airway macrophage subsets and transcriptomic changes were driven by cross-talk with airway epithelial cell populations, we performed CellChat [26] analysis. This tool facilitates inference and analysis of global cell–cell communication using gene expression profiles from scRNA-seq data and has been used in other lung studies [41, 42]. We discovered that the total number of inferred cell–cell interactions and interaction strength (determined by ligand–receptor gene expression) was markedly increased in IPF compared to healthy controls (figure 6a). Next, we quantified the differential strength of cell–cell interactions in the communication network, comparing IPF and healthy controls (figure 6b). In IPF compared to controls, the strongest interactions predicted were between airway macrophages and multiciliated cells (figure 6b). We then identified dominant signalling changes in “sender” and “receiver” cell populations in IPF compared to control (figure 6c). This showed stronger bidirectional signalling between airway macrophages and multiciliated cells in IPF. There was also evidence of airway macrophage autocrine signalling and increased signalling between airway macrophages (sender) and CD8 T-cells (receiver), club cells (receiver) and monocyte-derived macrophages (receiver). Comparing outgoing and incoming interaction strength in a two-dimensional scatter plot we were able to identify airway macrophages, and to a lesser extent monocyte-derived macrophages, as having the most significant changes in signalling between healthy controls and IPF patients (figure 6d). We found similar cell–cell interaction patterns when we performed a male-only CellChat analysis (supplementary figures S7 and S8).

In summary, CellChat captured a unique pattern of cell–cell communication within the proximal airway microenvironment in IPF with increased cross-talk between airway macrophages and multiciliated epithelial cells.

### Dysregulated signalling pathways between multiciliated cells and airway macrophages in IPF

**FIGURE 7:**
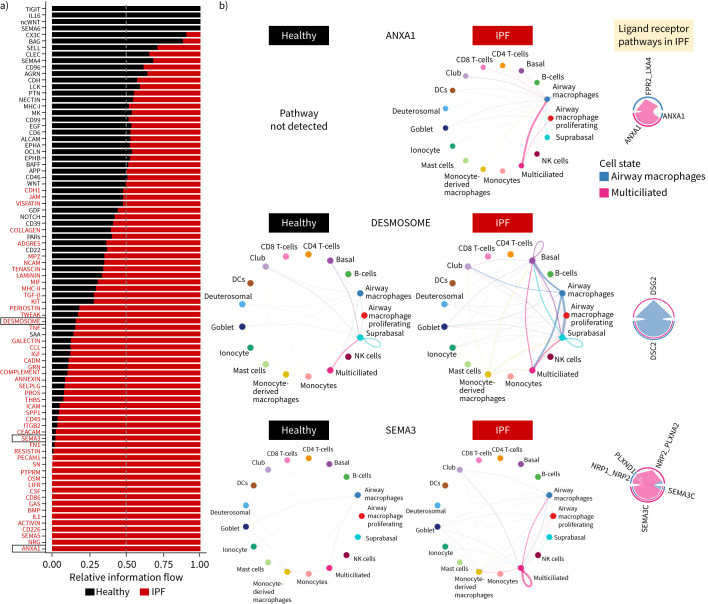
Dysregulated signalling pathways between multiciliated cells and macrophages in airways of idiopathic pulmonary fibrosis (IPF) patients compared to healthy controls. a) Significant signalling pathways were ranked based on differences in the overall information flow within the inferred networks between IPF and healthy controls. The overall information flow is calculated by summarising all communication probabilities in that network. The signalling pathways coloured red are enriched in IPF, and those coloured black are enriched in healthy controls. b) Circle plots showing selected signalling networks (ANXA1, DESOMOME and SEMA3) which were enriched in IPF relative to healthy controls. The weight of edges represents the difference in interaction strength. The colour represents the cellular source of the signalling. Corresponding ligand–receptor pair between airway macrophages and multiciliated cells for each signalling pathway shown as a chord diagram on the right-hand side. DC: dendritic cell; NK: natural killer.

Having identified the major interacting cell types in the airway, we sought to expose altered signalling pathways by comparing overall “information flow”; the sum of the communication probability among all pairs of cell groups in the inferred network for each signalling pathway, using CellChat [26]. Significant pathways were ranked based on information flow in IPF compared to controls (figure 7a). An identical approach was taken in the male-only analysis (supplementary figure S8). At a more granular level we were also able to compare outgoing and incoming signalling patterns in healthy controls and IPF patients (supplementary figure S9).

Among the top enriched signalling pathways in IPF were those previously linked to IPF pathogenesis including TGFB and SPP1 [14] (figure 7a). The top ranked pathway in IPF was ANXA1, comprising annexin A1 and its receptor, formyl peptide receptor 2 (FPR2). ANXA1 has been reported to play a role in monocyte chemotaxis [43] and fibrosis [44]. While not detected in healthy controls, there was increased ANXA1 signalling in IPF between *ANXA1* from multiciliated cells and *FPR2* on airway macrophages (figure 7b). We then focused on pathways in which the dominant interactions were between multiciliated cells and airway macrophages. There was an increase in DESMOSOME cell–cell adhesion network interactions between airway macrophages and epithelial cells notably multiciliated, suprabasal, club and basal cells, *via* DSC2 to DSG2. Increased cell–cell adhesion between multiciliated and suprabasal epithelial cells and basal epithelial cells was inferred. Cell–cell signalling pathways potentially driving proliferation of airway macrophages and monocyte-derived macrophages in IPF compared to healthy controls, include macrophage migration inhibitory factor (MIF) [45] signalling (supplementary figure S10A) from multiciliated cells to airway macrophages, and autocrine colony stimulating factor (CSF) [46] signalling (supplementary figure S10B). Strikingly, there was increased signalling in the SEMA3 (class 3 semaphorin) signalling pathway in IPF with multiciliated cells expressing *SEMA3C* sending signals to NRP1_NRP2 receptors on airway macrophages and also back to the multiciliated cells themselves (figure 7b). Proliferating airway macrophages also appear to contribute to SEMA3 signalling in IPF. SEMA3, a member of the semaphorin family, was originally described as a neuronal guidance protein, but semaphorins are now recognised to regulate numerous cellular processes including those linked to inflammation and remodelling in murine and human lungs during disease, but there is a paucity of data in IPF [47]. To determine the spatial context of the airway cell populations implicated in paracrine SEMA3 signalling, we performed imaging mass cytometry of IPF lung tissue sections. Airway macrophages expressing the surface marker CD68 [48] lined the airway lumen adjacent to E-cadherin-expressing multiciliated epithelial cells (supplementary figure S11A). Immunofluorescence microscopy of lung tissue sections demonstrated an increased SEMA3A signal in airway epithelial cells and endothelial cells (supplementary figure S11B).

In summary, we have discovered a novel and complex dialogue between epithelial cell subsets and airway macrophages in the airway niche of IPF patients. Specifically, there are marked changes in the SEMA3 epithelial–macrophage signalling network exclusive to the development of IPF.

## Discussion

A comprehensive single-cell analysis of nonmanipulated airway brushings to reveal both epithelial and immune cell populations from the proximal airway of IPF patients has not been reported. Herein we describe a scRNA-seq study of airway brushings from patients with IPF as compared to healthy controls, focussing on early- rather than late-stage disease, before antifibrotic treatment or need for long-term oxygen. We show a relative increase in abundance of macrophage subsets in the airway mucosal wall of IPF patients and changes in epithelial and immune cell populations at the level of gene expression and cell–cell communication. We reveal novel aberrant interactions and signalling networks between multiciliated epithelial cells and airway macrophages, in proximal airway mucosa in patients newly diagnosed with IPF as compared to healthy controls. Perturbations in airway macrophage subsets correlated with disease extent and disease progression and thus may provide avenues for exploring improved diagnostic strategies and airway-targeted interventions such as inhaled therapies which may be more efficacious in early-stage disease.

Greater accessibility to explant lung tissue samples from diseased donors has fuelled studies of the distal lung environment in advanced IPF [10, 11], rather than the proximal airway niche earlier in disease. The association of the airway mucin *MUC5B* variant rs35705950 with disease susceptibility [4], and the expansion of basal cell epithelial populations in the distal lung [49], have ignited interest in the potential role of airway cell populations in this devastating disease [50]. To date, studies of the IPF airway niche have focussed on changes in epithelial cell populations [13, 17]. Indirect evidence suggesting an imbalance in airway mucosal immune homeostasis in IPF has arisen from BAL studies reporting an altered microbiome and significantly increased bacterial burden in disease [51–53]. Our in-depth analysis using nonmanipulated proximal airway brushings reveals that the mucosal airway niche is a heterogeneous community, consisting of epithelial cells coexisting with a significant immune cell component. Increased gene expression in adhesion pathways in this mucosal niche indicates strong interactions between immune cells and the epithelial lining cells.

Macrophages are the most abundant immune cells in the airspace, and are strategically positioned to facilitate vital immune surveillance, process surfactant, and clear apoptotic debris [54]. scRNA-seq of digested lung tissue from IPF patients exposed aberrant macrophage populations in the alveolar and interstitial regions [10, 11, 14]. Our study is the first to report compositional and transcriptomic differences in airway macrophage communities lining the proximal airway mucosa in newly diagnosed cases of IPF. Macrophage phenotype depends on signals from the microenvironmental niche [55]. We identified three key macrophage populations: airway macrophages, monocyte-derived macrophages, and proliferating airway macrophages. It is likely that these subsets represent distinct phases of monocyte-to-macrophage differentiation as described in scRNA-seq studies of human BAL in health [56] and disease states [35, 57]. Notably, these macrophage subsets were increased in IPF compared to healthy controls, and we identified alterations in MIF and CSF cell–cell signalling pathway networks as potential drivers of increased macrophage proliferation. Increased peripheral blood monocyte counts have been associated with increased mortality in IPF [58]. We found *MCEMP1* (mast-cell expressed membrane protein-1), a gene predictive of poor outcome in IPF [59, 60] and previously found to be expressed in IPF alveolar macrophages [61], was one of the top DEGs showing increased expression in IPF airways macrophages compared to healthy controls. Proliferating airway macrophages expressing cell cycle markers appeared to be intrinsically linked to the pathogenesis of IPF as were absent in all but one healthy control. Higher airway macrophage and proliferating airway macrophage abundance in IPF was associated with lower lung function and greater radiological extent of fibrosis at baseline. An IPF-specific population of profibrotic macrophages expressing *SPP1*, a gene encoding osteopontin, has been reported in diseased lung tissue [10, 11, 14]. Osteopontin is a secreted glycoprotein that drives proliferation and migration of human monocyte-derived macrophages [62]. SPP1 has been localised to alveolar epithelial cells in IPF lungs and was elevated in BAL fluid [63]. We discovered higher expression of *SPP1* in monocyte-derived macrophages, a population that was increased in IPF relative to controls and associated with disease progression at 12 months. Additionally, the SPP1 signalling pathway was enriched in IPF compared to healthy controls.

We found immune cell populations on nonmanipulated airway brushings following BAL, implying strong adhesive cell interactions with the epithelium. In a mouse model, the immune response to inhaled lipopolysaccharide was modulated by cross-talk *via* connexin 43-containing gap junctions between resident alveolar macrophages and alveolar epithelial cells [64]. However, these direct interactions have yet to be explored in the human airway, particularly in the proximal airways. To investigate cellular interactions at the airway wall, communicating pairs of cells were predicted by the expression of interacting ligands and receptors, using CellChat [26]. This computational approach identified an increase in the DESMOSOME cell adhesion network between airway macrophages and multiciliated cells in IPF compared to control. Although DSC2-DSG2 have been shown to mediate interactions between macrophages and the epithelium in the colonic mucosa [65], this is the first description of a direct interaction in the IPF airway. Genome-wide association studies have implicated cell–cell adhesion pathways in IPF susceptibility, including a genetic variant rs2076295 in the *DSP* gene*,* encoding desmoplakin, a component of the desmosome [66].

Semaphorin signalling was notably dysregulated in the IPF airway niche. Semaphorins provide axonal guidance cues during neuronal developmental, but are also involved in distal pulmonary epithelial cell development [67] and immunoregulation [68]. Semaphorin biology has not been studied extensively in the lung or IPF [47], although SEMA4 [69] and SEMA7 [70] have been linked to development of IPF. An interactome analysis using single-cell data from a human airway epithelial–lung fibroblast co-culture model has suggested a role of semaphorin-mediated signalling *via* plexin receptors [71]. Spatial transcriptomic analysis of FFPE lung tissue from three IPF patients compared to four controls, reported SEMA3 as a prevalent secreted signalling pathway in the fibrotic niche composed of aberrant basaloid cells and myofibroblasts located around airways [41]. Our study is the first to report a key role for SEMA3 in airway epithelial–macrophage interactions in newly diagnosed cases of IPF.

Our study had several limitations. First, due to the nature of working with fresh (rather than fixed) proximal airway brushing samples, the study sample size was relatively small and therefore the findings require confirmation in a larger cohort to account for interindividual variability. Second, all IPF cases recruited to our study were male, due to the higher incidence of IPF in males compared to females. To address the single-sex issue in our study, we reproduced our original findings using a male-only analysis of our IPF samples *versus* healthy controls, demonstrating that the findings of our study are robust despite the sex imbalance. Although single-cell analysis has shown that sex can cause differences in cellular transcriptional phenotype, the Human Lung Cell Atlas analysis [22] reported that effects of sex on gene expression were largely restricted to lymphatic endothelial cells, which are not relevant in our dataset. Further validation in a larger cohort of sex-matched IPF patients and healthy controls is required. Thirdly, the cross-sectional nature of our study precludes conclusions about a direct causal relationship between airway macrophage subsets and disease progression and warrants further investigation with longitudinal sampling.

Since the proximal airway represents an unexplored niche in IPF, it is not possible to directly compare our dataset with existing IPF single-cell atlases of the distal lung, which is anatomically distinct. The cell–cell interaction findings generated by the CellChat analysis are predictions based on computational inference. These novel data are hypothesis-generating and future work will focus on functional validation *in vitro* using co-culture models.

In summary, we have characterised the unique cellular ecosystem of the proximal airway mucosal wall in incident cases of IPF and exposed enhanced dialogue between multiciliated epithelial cells and airway macrophages which may be critical for the evolution and progression of disease. Our data suggest that immune dysregulation in the airway mucosal niche is in part orchestrated through direct interactions between airway macrophages and multiciliated cells and is intrinsically linked to the development of IPF.

## Supplementary material

**Please note:** supplementary material is not edited by the Editorial Office, and is uploaded as it has been supplied by the author. Supplementary methods 01273-2025.SUPPLEMENT Supplementary figures 01273-2025.SUPPLEMENT2 Table S1 01273-2025.SUPPLEMENT Table S4 01273-2025.SUPPLEMENT4 Table S5 01273-2025.SUPPLEMENT5 Table S6 01273-2025.SUPPLEMENT6 Table S7 01273-2025.SUPPLEMENT7 Table S2 01273-2025.SUPPLEMENT2 Table S3 01273-2025.SUPPLEMENT3
