# Supplementary material for: Single-cell RNA sequencing reveals aberrant airway epithelial–immune cell cross-talk in pulmonary fibrosis
Source: ERJ Open Res. 2026 Aug 3;12(4):01273-2025. doi: 10.1183/23120541.01273-2025 (PMC13430588; doi:10.1183/23120541.01273-2025)
Supplement: Supplementary file 1 [file 01273-2025.SUPPLEMENT.pdf]

Supplementary Methods

Cell isolation from airway brushings

Two bronchial brushings (Olympus) were taken from the right main bronchus during bronchoscopy and placed directly into Hank's Balance Salt Solution (HBSS, Gibco) supplemented with penicillin (1000 U/ml) and streptomycin (1mg/ml). We used a previously established method to isolate single- cells from airway brushings[1]. The brushes were centrifuged (1200rpm, 5mins, 4°C) and the resulting cell pellet resuspended in 1ml HBSS with 1mg/ml collagenase D from *Clostridium histolyticum* (Roche) and 0.1mg/ml DNase I from bovine pancreas (Roche) to and transferred to a FACS tube. The cell suspension was incubated for 1 hour at 37°C, with gentle agitation to avoid cell clumping. Dissociated cells were then passed through a 70μm filter, centrifuged (1200rpm, 5mins, 4°C) and the resulting cell pellet resuspended in HBSS with 0.04% UltraPure BSA (Invitrogen). The single-cell suspension was cleared of red blood cells using ACK red cell lysis buffer (Gibco). A final single-cell suspension of airway epithelial cells in 300μl HBSS with 0.04% UltraPure BSA was processed for scRNA-seq. The viability and concentration of each single-cell suspension was measured using trypan blue (Sigma) and the Eve automatic cell counter (NanoEntek).

Cytospins of airway brushings

To determine cellular constituents of a bronchial brush a cytospin was prepared. Cells were detached from the brush by agitation and centrifuged as previously described. The cells recovered were counted using a haemocytometer (~2.0–4.0 x 10^{4}), resuspended in PBS and centrifuged (18 *x g*, 4 minutes). The cells were fixed in 4% paraformaldehyde (PFA) (Electron Microscopy Sciences) for 20 minutes, washed in PBS, then permeabilised with permeabilisation buffer (eBioscience) for 20 minutes (room temperature). A PAP pen (Sigma) was used to demarcate the area on the slide to be stained. The cells were blocked with 2% BSA in PBS for 30 minutes (room temperature) then stained with primary antibodies to acetylated alpha-tubulin (Abcam #ab24610) and cytokeratin 5 (Abcam #ab193895), detailed in Table 2.2 for 2

hours at room temperature. A goat anti-mouse secondary antibody conjugated to Alexa Fluor 568 (1:200; Life technologies) was then added for 30 minutes (room temperature) and nuclei counterstained with DAPI (1:2000; Sigma-Aldrich). Mowiol 4-88 (Sigma-Aldrich) was used to mount the 13mm coverslip prior to imaging on Leica SP5 inverted confocal microscope equipped with four lasers (405nm, 488nm, 543nm, 633nm). Images were acquired using the LAS AF platform (Leica) and analysed using Fiji software **(http://fiji.sc**)[2].

Single-cell library construction and next-generation sequencing

Approximately 1.0 x 10^4 – 1.3 x 10^4 cells were loaded for each sample into a separate channel of a Chromium Chip G for use in the 10X Chromium Controller (#PN-1000120). The cells were partitioned into nanoliter scale Gel Beads in emulsions (GEMs) and lysed using the 10x Genomics Single Cell 3′ Chip v3.1 GEM, Library and Gel Bead Kit (#PN-1000121). cDNA synthesis and library construction were performed as per the manufacturer's instructions (Chromium Single Cell 3' Reagent Kits v3 User Guide). The RNA was reversed transcribed and amplified using 12 cycles of PCR. Libraries were prepared from 10µl of the cDNA and 12 cycles of amplification. Each library was prepared using Single Index Kit T Set A (#PN-1000213) and sequenced on the HiSeq4000 system (Illumina) using 100 bp paired-end run to an approximate depth of 50,000 reads per cell. Libraries were generated in independent runs for the different samples.

Single-cell RNA-seq data analysis

Raw data processing, QC and expression normalisation

Raw reads were initially processed by the Cell Ranger (10X Genomics) v3.0.2 pipeline, which deconvolved reads to their cell of origin using the UMI tags, aligned these to the human transcriptome (version GRCh38-3.0.0) using STAR (v2.5.1b) and reported cell-specific gene expression count estimates. Raw feature count matrix from the 'EmptyDrops' algorithm output of Cell Ranger were used to correct for Ambient RNA using the FastCAR[3] R package with default settings **(**https://github.com/LungCellAtlas/FastCAR**).**

All subsequent analyses were performed in R v3.6.0 using the Seurat (v3.0) package[4]. Genes were considered to be expressed if the estimated (log10) count was at least 0.1. Filtering was then performed to remove from consideration; cells with less than 1000 mapped reads; cells expressing fewer than 500 genes and cells for which mitochondrial genes made up greater than 3 standard deviations from the median per patient. Each dataset was normalised using the 'logNormalize' function with a scale factor of 10,000.

The top 2000 highly variable genes were found using the 'FindVariableGenes' function and the data centred and scaled using the 'ScaleData' function. PCA decomposition was performed and, after consideration of the eigenvalue 'elbow-plots', the first 40 components were used to construct the UMAP plot for each individual sample. Doublets were flagged using the DoubletFinder package[5], and rates calculated per sample, but not removed from downstream analyses.

Dimensionality reduction and visualisation

Samples from IPF and healthy controls were integrated using Seurat's 'IntegrateData' function using 2000 integration anchors and the first 50 components were used to construct the integrated UMAP plot. To evaluate the contribution of each subject to the integrated clusters obtained, a UMAP was generated for each sample using the integrated UMAP 'space'.

Cell type abundance

We used three methods for cell type abundance testing:

1) To compare cell type abundance between IPF and healthy controls (Figure 3C and 3E) a Mann-Whitney test and the Benjamini and Hochberg FDR method of multiple comparison correction was used with a desired FDR (Q) 1%. Those with a q value (FDR-adjusted p value) <0.05 are indicated on the figure; an asterisk represents a "discovery" q value <0.01, while "nd" represents "not a discovery" q ≥0.01.
2) To determine changes in cell type abundance between IPF and healthy control samples the miloR package[6] https://github.com/MarioniLab/miloR was

used (Figure 3F). Cells were divided into cell neighbourhoods using K nearest neighbours graph-based reduction method using default parameters. Neighbourhood testing was performed to test for differential abundance between IPF versus healthy controls by fitting a quasi-likelihood negative binomial generalized model to count data using the function glmQLFit and using the Quasi-Likelihood F-test. P values were corrected for with the Benjamini-Hochberg method. Neighbourhoods were annotated based on > 70% cell type assignment. A 'Mixed' annotation was assigned to neighbourhoods where there were no single-cell type >70%. Results were visualised using ggplots.

3) Cluster abundance testing for condition, smoking status, progression status and survival status (Supplementary Table 3) was determined using negative binomial general linear methods from the edgeR package[7] (version 4.4.2), using patient samples as replicates.

Differential gene expression analysis

Differentially expressed genes (DEGs) between IPF progressors versus IPF non-progressors (Supplementary Table 4) and IPF ex-smokers versus IPF never smokers (Supplementary Table 5) were identified using the package glmGlamPoi[8] (version 1.18) for all cell-types in pseudo-bulk manner. Expression profiles were aggregated into pseudobulk expression profiles for each cell type of interest and patient. glmGlamPoi works by implementing a generalised log-linear regression model against a negative-binomial distribution to model the effects of disease groups on cell counts, using patient sample as replicates. Results were filtered for 10 > log2 fold change > 0.25 (absolute value) and adjusted p value (Bonferroni correction) < 0.05.

Cell- cell interaction analysis

Cell-cell interactions were assessed using CellChat[9] (version 1.1.3) running on R version 4.1.0. To identify differential regulated signalling pathways, grouped Healthy and IPF sample datasets were separated and analysed in parallel using the default

parameters, with the ‘population.size’ parameter set to “TRUE” when computing the communication probability.

Imaging Mass Cytometry (IMC)

An antibody panel was designed to capture epithelial cells and macrophages in the airway microenvironment. We used the following pre-chelated antibodies: anti-human collagen type 1 (Polyclonal)- 169Tm (Fluidigm, #3169023D), anti-human CD45 (D9M8I) – 152Sm (Fluidigm, #3152018D), anti-human smooth muscle actin (1A4) – 141Pr (Fluidigm, #3141017D), anti-human E-Cadherin (24E11) – 158Gd (Fluidigm, #3158029D) and anti-human CD68 (KP1) – 159Tb (Fluidigm, #3159035D). Each antibody was tested on FFPE tissue sections from controls and patients with IPF to ensure an expected staining pattern and optimal dilution with good signal-to-noise ratio for each channel. The dilution factor was altered for channels with obvious spillover into neighbouring channels.

FFPE lung tissue sections (4μm thickness) from controls and patients with IPF were stained with H&E and imaged on Aperio Versa Slide Scanner (Leica). Tissue integrity and suitability for IMC was assessed in Aperio ImageScope (Leica). Suitable serial FFPE lung tissue sections were dewaxed by baking in a dry oven at 60°C for 2 hours. The sections were placed in Gentaclear (Genta Medical) for 2 x 10 minutes, followed by rehydration in a graded series of ethanol (100%, 95%, 80%, 70%) for 5 minutes each. Sections were washed on a shaker in Maxpar water (Standard BioTools Inc.) for 5 minutes. Heat-induced antigen retrieval was performed at 96°C in Dako Target Retrieval Solution (Agilent; diluted from 10x to 1x) for 30 minutes. After cooling to less than 70°C, sections were washed with Maxpar water and Maxpar phosphate-buffered saline (PBS; Standard BioTools Inc). The sections were blocked with 3% Bovine serum albumin (BSA; Sigma-Aldrich) in Maxpar PBS (Standard BioTools Inc) for 45 minutes at room temperature (RT). Sections were incubated at 4°C overnight with primary antibodies in 0.5% BSA (Table 2). Slides were washed x2 for 8 minutes in 0.2% Triton X-100 (Sigma-Aldrich) in Maxpar PBS (Standard BioTools Inc), followed by 2 washes in Maxpar PBS (8 minutes per wash). Sections were counterstained with CELL-ID intercalators (Ir; Standard BioTools Inc),

added to each section and incubated at RT for 30 minutes. Sections were washed in Maxpar water for 5 minutes and air-dried for 30 minutes. The slides were stored without coverslips at RT until imaging. The Hyperion Imaging System (Standard BioTools Inc) was used to attain IMC images. Following start-up in CyTOF 7.0 (Standard BioTools Inc), the system was calibrated using a 3-element full coverage tuning slide (Standard BioTools Inc) which utilises a polymer matrix containing Yttrium-89, Cerium-140 and Lutetium-175. On completion of successful calibration, simple brightfield images of the tissue were taken using the 'Panorama' function, Regions of interest (ROIs) were selected within areas of AR. Laser ablation was performed with laser power 2, frequency 200 Hz and 1μm resolution. Raw data were exported from CyTOF 7.0 in .mcd format. MCD Viewer (Standard BioTools Inc) was used to review each channel to ascertain image quality and pseudo-colours were assigned to channels of interest to generate a composite image which was exported as a .tiff file.

Immunofluorescence microscopy

Immunofluorescence microscopy was used to determine the localisation of semahorin-3A protein expression in human lung tissue. FFPE lung tissue sections (4μm thickness) from controls and patients with IPF were baked for 2 hours at 60°C to melt the paraffin. Slides were then dewaxed in Gentaclear (Genta Medical) for 2x 10 minutes, followed by rehydration in a graded series of ethanol (100%, 90% and 70%) for 5 minutes each. Slides were then gently washed in demineralised (DM) water for 5 minutes on a shaker. Antigen retrieval is performed in freshly made Tris-EDTA buffer (pH 9.0) that is pre-heated in a microwave and then adjusted to ~96°C in a rice cooker (Cookworks, #724/8236). The slides were incubated in the antigen retrieval buffer at 96°C for 30 minutes. Slides were then cooled to 70°C and washed in 1x in DM water and 1x in PBS on a shaker for 5 minutes each. Slides were blocked in animal-free blocker (Vector Laboratories, #SP-5030-250) for 45 minutes at RT. Slides were incubated overnight at 4°C in SEMA3a primary antibody (1:50, Abcam, #ab199475) in animal-free blocker. Slides were washed x2 in 0.5% PBS-Tween on a shaker for 5 minutes each. The slides were then incubated at RT for 2 hours in the goat anti-rabbit IgG Alexa Fluor^{TM} 594 secondary antibody (1:200,

ThermoFisher Scientific, #A-11012). After incubation, slides were washed 2x 5 minutes in 0.5% PBS-Tween, then 2x 5 minutes in PBS. Slides were counterstained with 4′,6-diamidino-2-phenylindole (DAPI, Sigma-Aldrich) at 1:2,000 for 5 minutes at RT. Slides were washed 2x in PBS for 5 minutes each on a shaker. Slides were then mounted and coverslipped with Prolong™ Diamond antifade mountant (Invitrogen, #P36961). Imaging was conducted on a Leica Stellaris 5 inverted point scanning confocal microscope witha Plan Apo CS2 20x/0.75 objective (Leica, #11506517), with a zoom of 1 and 4096x4096 pixels in the frame, giving a frame size of 581 microns and a pixel size of 142 nanometres, and a line averaging of 4. DNA was stained with DAPI and imaged with a 405nm laser and a HyD S detector (425nm-504nm), and SEMA3 imaged with a White Light Laser set at 557nm and a HyD S detector (562nm-721nm). Panel was prepared with Fiji.

Statistical methods

Statistical analysis was carried out using Prism v9.4.1 (GraphPad Software, LLC.). To determine if the data was from a Gaussian distribution the D'Agostino-Pearson omnibus normality test was performed. If the p- value returned for this test was high (>0.05), it was assumed the data were sampled from a Gaussian distribution and a parametric test was used for statistical analysis; for unpaired data this was an unpaired t test, and for paired data this was a paired t-test. If the normality test p-value was ≤0.05, the null hypothesis was rejected and therefore data were not sampled from a Gaussian distribution so a nonparametric test was used; for unpaired data this was a Mann-Whitney test and for paired data this was a Wilcoxon matched-pairs signed rank test. For correlation analyses, Pearson correlation coefficients were computed and a two-tailed P value, ≤0.05 considered significant. The statistical test used for each analysis are described in the accompanying figure legend and significant differences noted by asterisk(s): *p<0.05, **p<0.01, ***p<0.001, ****p<0.0001.

Data availability

Raw data has been submitted to NCBI's GEO repository under the accession ID GSE275264.

References

1 Vieira Braga, F. A. *et al.* A cellular census of human lungs identifies novel cell states in health and in asthma. *Nat Med* **25**, 1153-1163 (2019). https://doi.org/10.1038/s41591-019-0468-5

2 Schindelin, J. *et al.* Fiji: an open-source platform for biological-image analysis. *Nat Methods* **9**, 676-682 (2012). https://doi.org/10.1038/nmeth.2019

3 Berg, M. *et al.* FastCAR: Fast Correction for Ambient RNA to facilitate differential gene expression analysis in single-cell RNA-sequencing datasets. *bioRxiv*, 2022.2007.2019.500594 (2022). https://doi.org/10.1101/2022.07.19.500594

4 Stuart, T. *et al.* Comprehensive Integration of Single-Cell Data. *Cell* **177**, 1888-1902.e1821 (2019). https://doi.org/10.1016/j.cell.2019.05.031

5 McGinnis, C. S., Murrow, L. M. & Gartner, Z. J. DoubletFinder: Doublet Detection in Single-Cell RNA Sequencing Data Using Artificial Nearest Neighbors. *Cell Syst* **8**, 329-337.e324 (2019). https://doi.org/10.1016/j.cels.2019.03.003

6 Dann, E., Henderson, N. C., Teichmann, S. A., Morgan, M. D. & Marioni, J. C. Differential abundance testing on single-cell data using k-nearest neighbor graphs. *Nature Biotechnology* **40**, 245-253 (2022). https://doi.org/10.1038/s41587-021-01033-z

7 Robinson, M. D., McCarthy, D. J. & Smyth, G. K. edgeR: a Bioconductor package for differential expression analysis of digital gene expression data. *Bioinformatics* **26**, 139-140 (2009). https://doi.org/10.1093/bioinformatics/btp616

8 Ahlmann-Eltze, C. & Huber, W. glmGamPoi: fitting Gamma-Poisson generalized linear models on single cell count data. *Bioinformatics* **36**, 5701-5702 (2020). https://doi.org/10.1093/bioinformatics/btaa1009

9 Jin, S. *et al.* Inference and analysis of cell-cell communication using CellChat. *Nature communications* **12**, 1088 (2021). https://doi.org/10.1038/s41467-021-21246-9
