# Supplementary material for: Single-cell RNA sequencing reveals aberrant airway epithelial–immune cell cross-talk in pulmonary fibrosis
Source: ERJ Open Res. 2026 Aug 3;12(4):01273-2025. doi: 10.1183/23120541.01273-2025 (PMC13430588; doi:10.1183/23120541.01273-2025)
Supplement: Supplementary file 2 [file 01273-2025.SUPPLEMENT2.pdf]

Single-Cell RNA-seq Reveals Aberrant Airway Epithelial-Immune Cell Crosstalk In Pulmonary Fibrosis

Richard J. Hewitt, Probir Chakravarty, Jimena Perez-Lloret, Martin Banchero, Marijn Berg, Maarten van den Berge, William J. Traves, Laura L. Yates, Simone A. Walker, David C. A. Gaboriau, Alexandra Rice, Andrew G. Nicholson, Anand Devaraj, Samuel V. Kemp, Philip L. Molyneaux, Franz Puttur, Adam J. Byrne, Toby M. Maher, Martijn C. Nawijn, Anne O'Garra and Clare M. Lloyd

Supplementary Figures

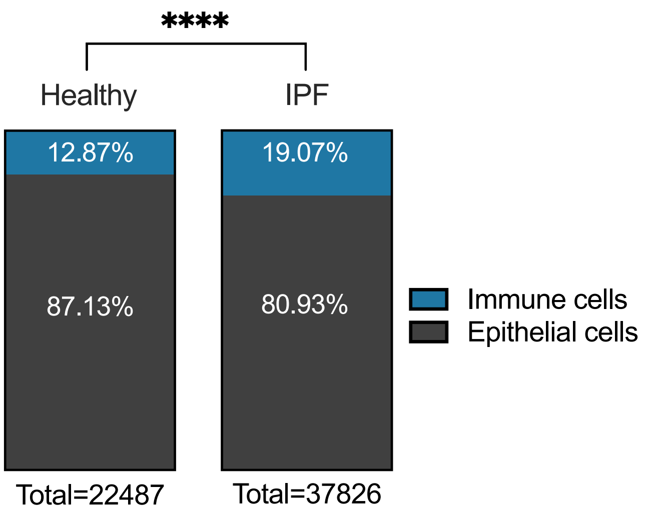

Supplementary Figure 1. Proportion of epithelial cells and immune cells on non-manipulated airway brushings from IPF patients and healthy controls. Contingency analysis of cell proportions according to cell lineage. Fisher's exact test, two-sided ****p<0.0001.

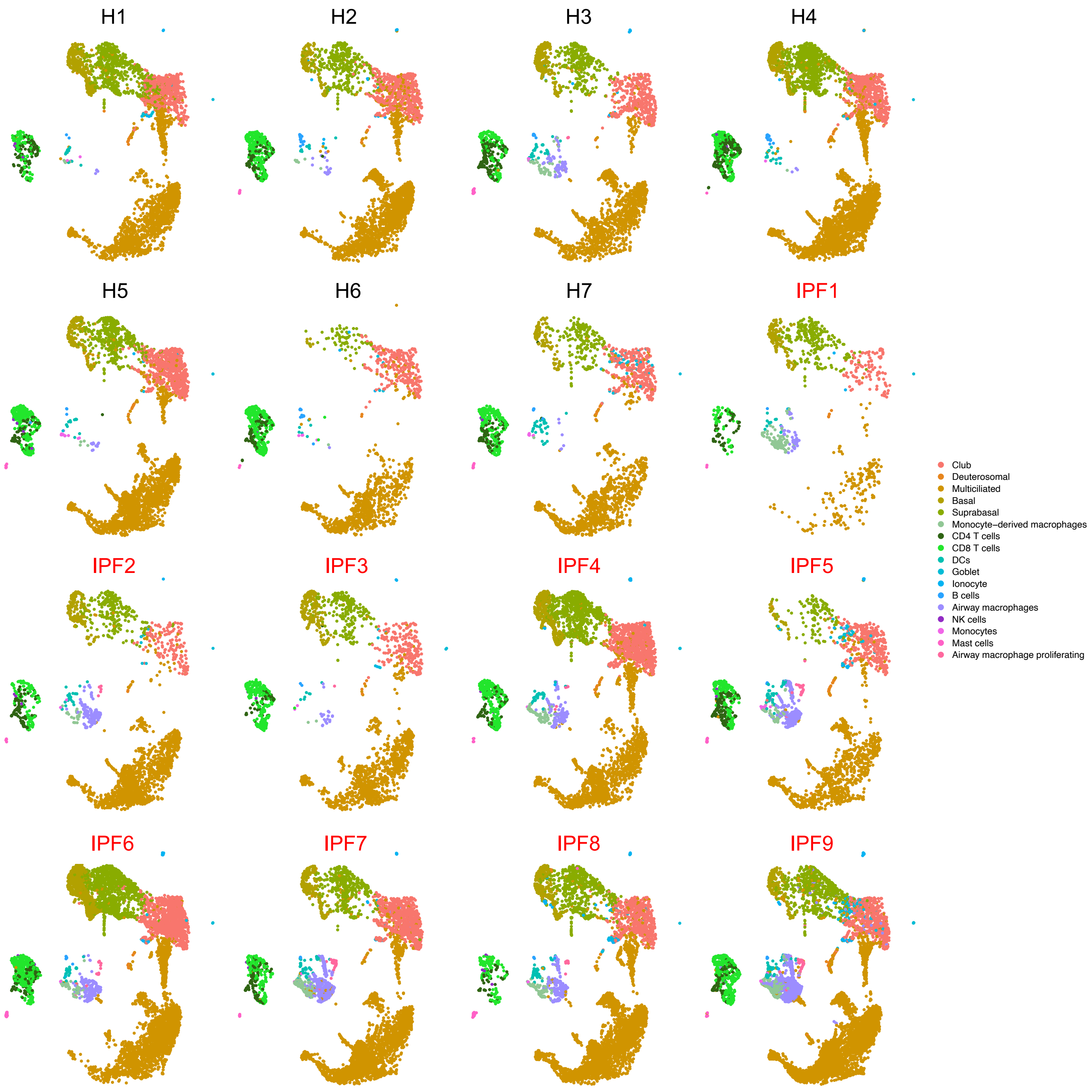

Supplementary Figure 2. Uniform manifold approximation and projection (UMAP) representations of cells from airway brushings per subject. UMAPs from individual healthy subjects labelled H1 – H7 and from IPF patients labelled IPF1 – IPF9. Colour specifies assignment of cells to one of 17 clusters with labels transferred from Level 5 annotation of the Human Lung Cell Atlas.

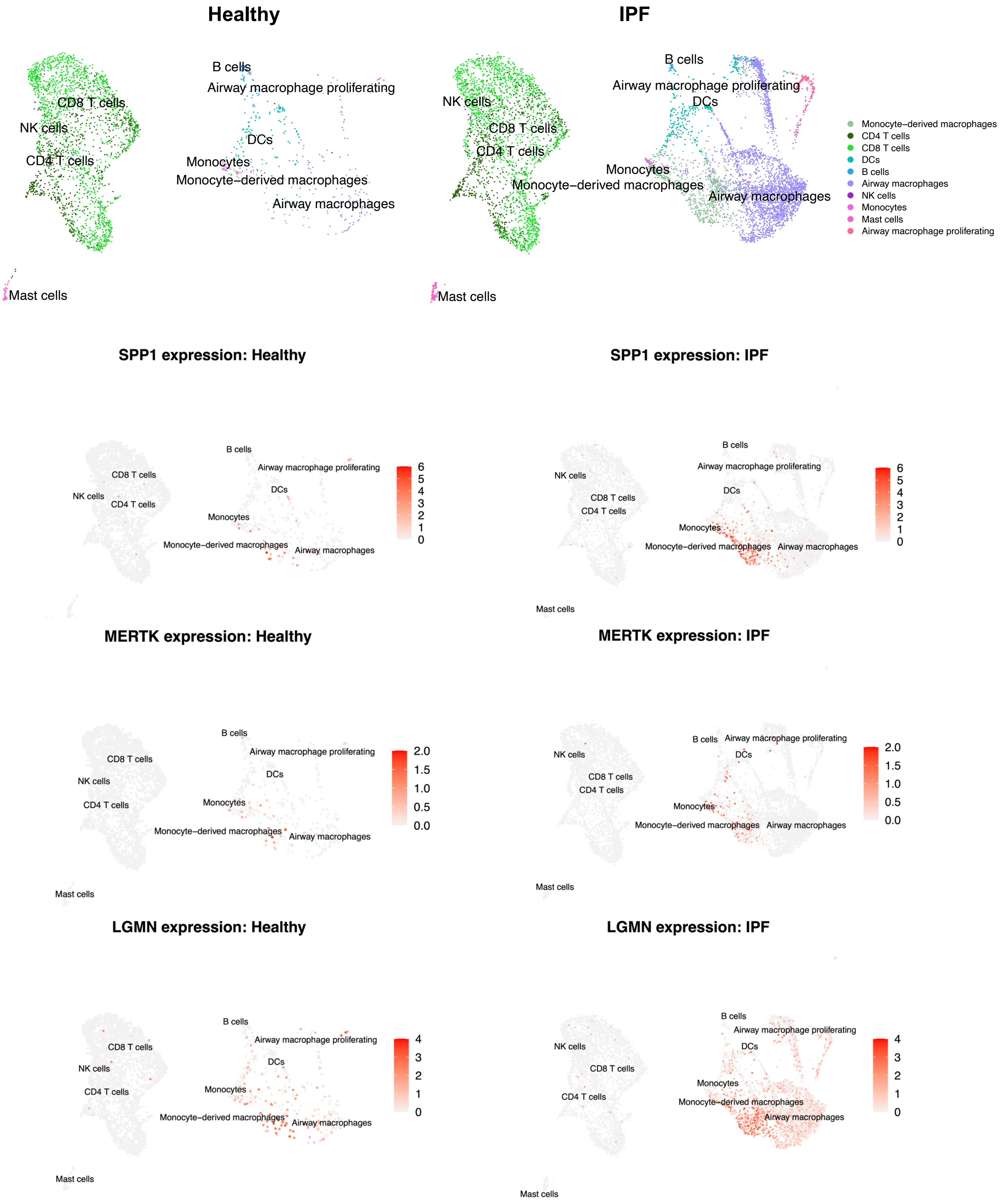

Supplementary Figure 3. Identification of *SPP1*^hi macrophage subpopulation in IPF airway brushings compared to healthy controls. UMAP visualisation of immune cell clusters in airway brushings from healthy controls and IPF patients (top panel). Feature plots showing expression of *SPP1* and other genes known to be co-expressed with this profibrotic macrophage subset; *MERTK* and *LGMN.*

Male – only analysis

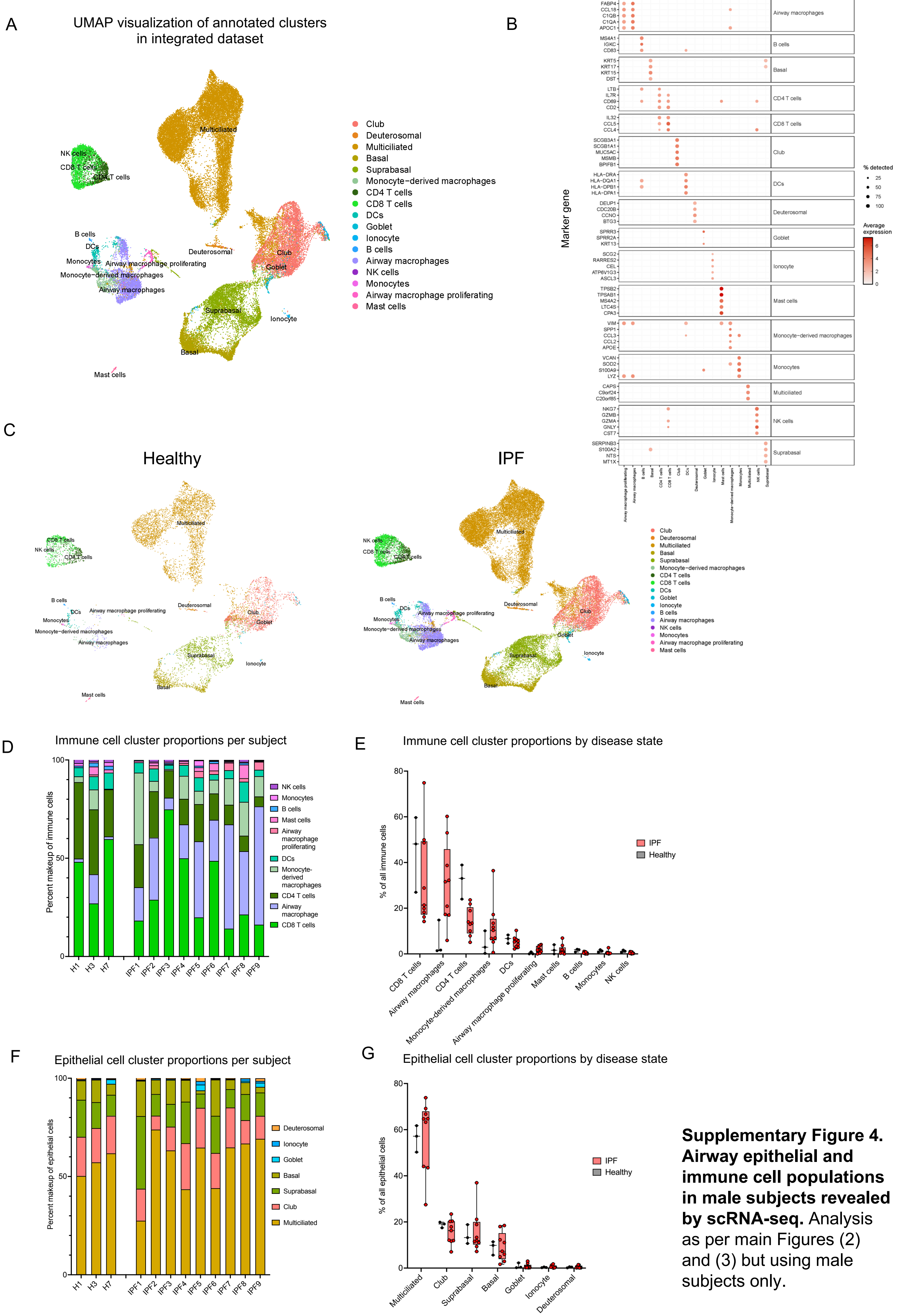

Supplementary Figure 4. Airway epithelial and immune cell populations in male subjects revealed by scRNA-seq. Analysis as per main Figures (2) and (3) but using male subjects only.

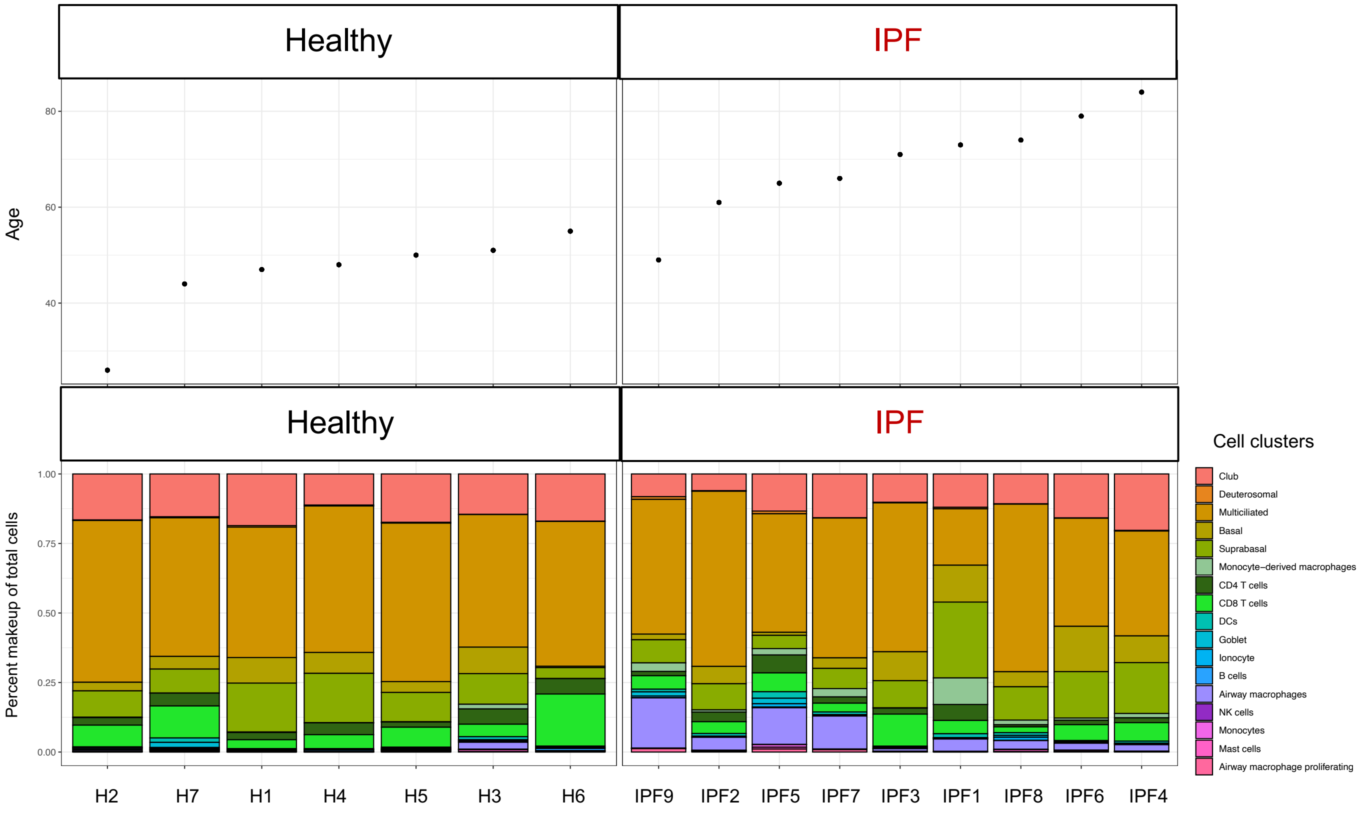

Supplementary Figure 5. The effect of age on airway epithelial and immune cell populations in IPF and healthy controls. Bar chart showing cell type proportions per subject in order of advancing age for healthy controls and IPF patients.

Male – only analysis

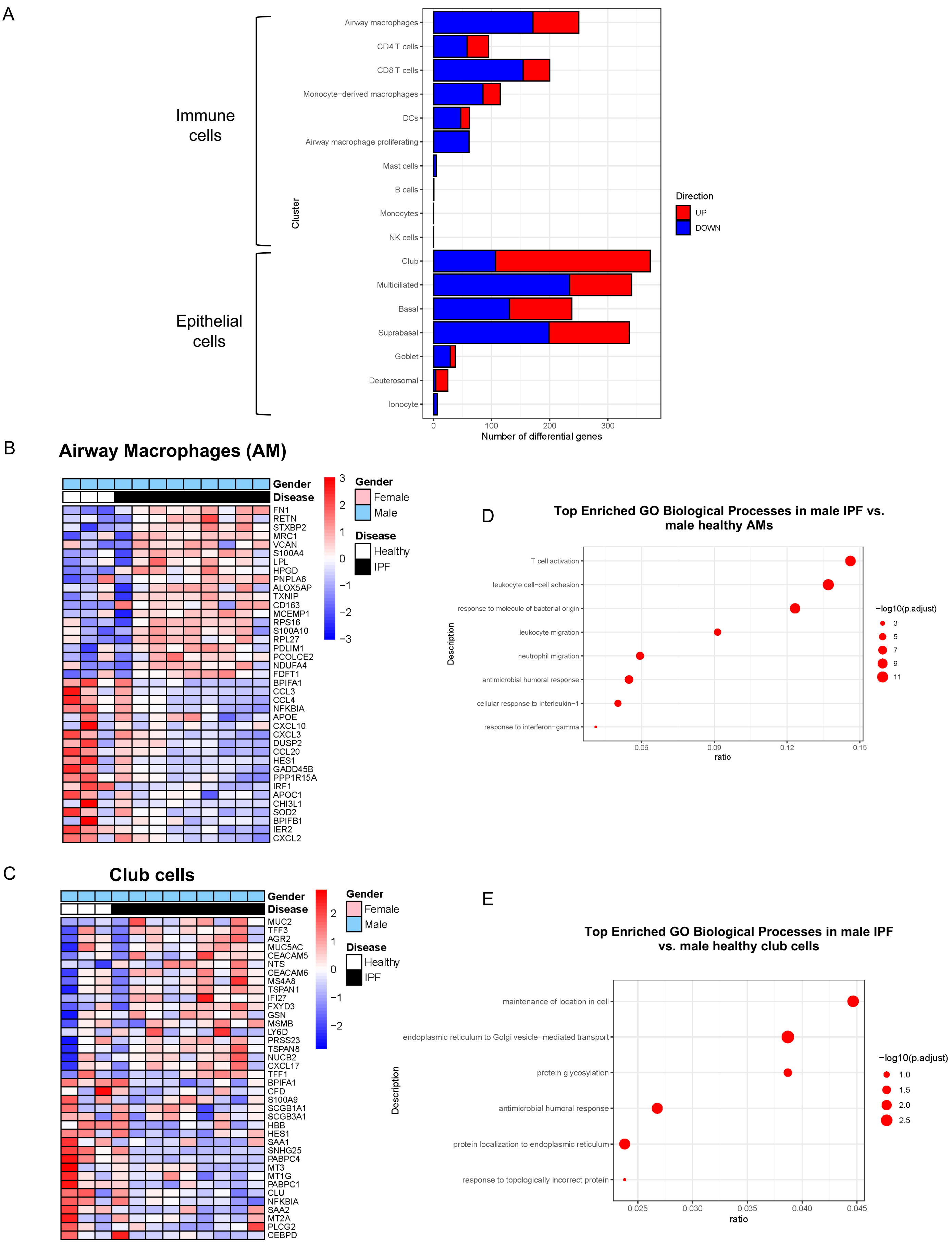

Supplementary Figure 6. Transcriptomic changes in airway epithelial and immune cell populations in male only-subjects. (*A*) Number of differentially expressed genes comparing male IPF patients with male healthy controls for each cell type (log fold change (FC) cutoff of 0.25 and adjusted P value < 0.05). In Figures E5 (B) and (C) we have shown the expression of the top genes from the original analysis in the male-only dataset and in (D) and (E) we have taken selected pathways from the original analysis and tested them in the male-only analysis.

Male – only analysis

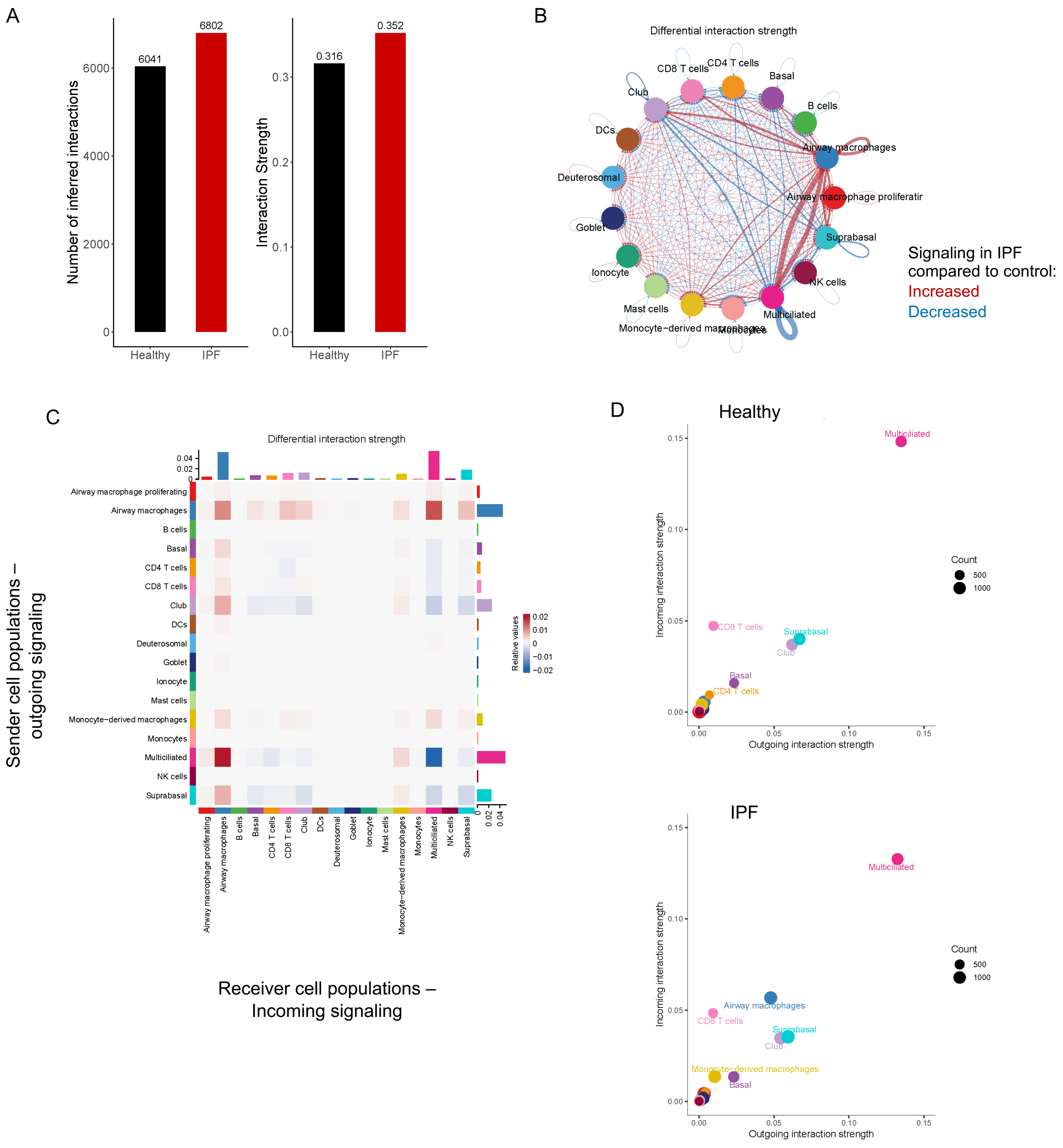

Supplementary Figure 7. Increased cell-cell interactions in the airways of male IPF patients compared to male healthy controls. Analysis as per main Figure 6 but using male subjects only.

Male – only analysis

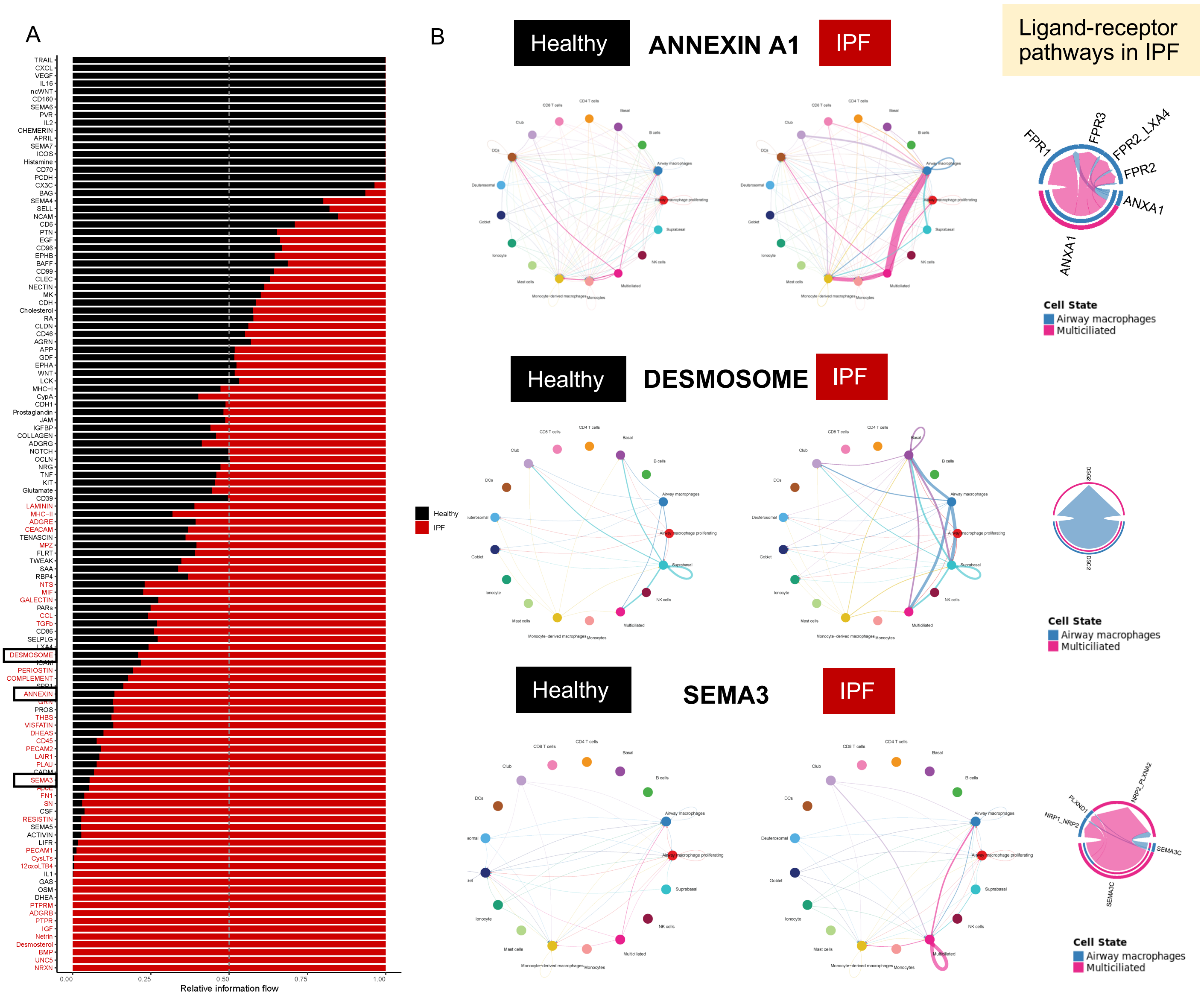

Supplementary Figure 8. Dysregulated signaling pathways between multiciliated cells and macrophages in airways of male IPF patients compared to male healthy controls. Analysis as per main Figure 7 but using male subjects only.

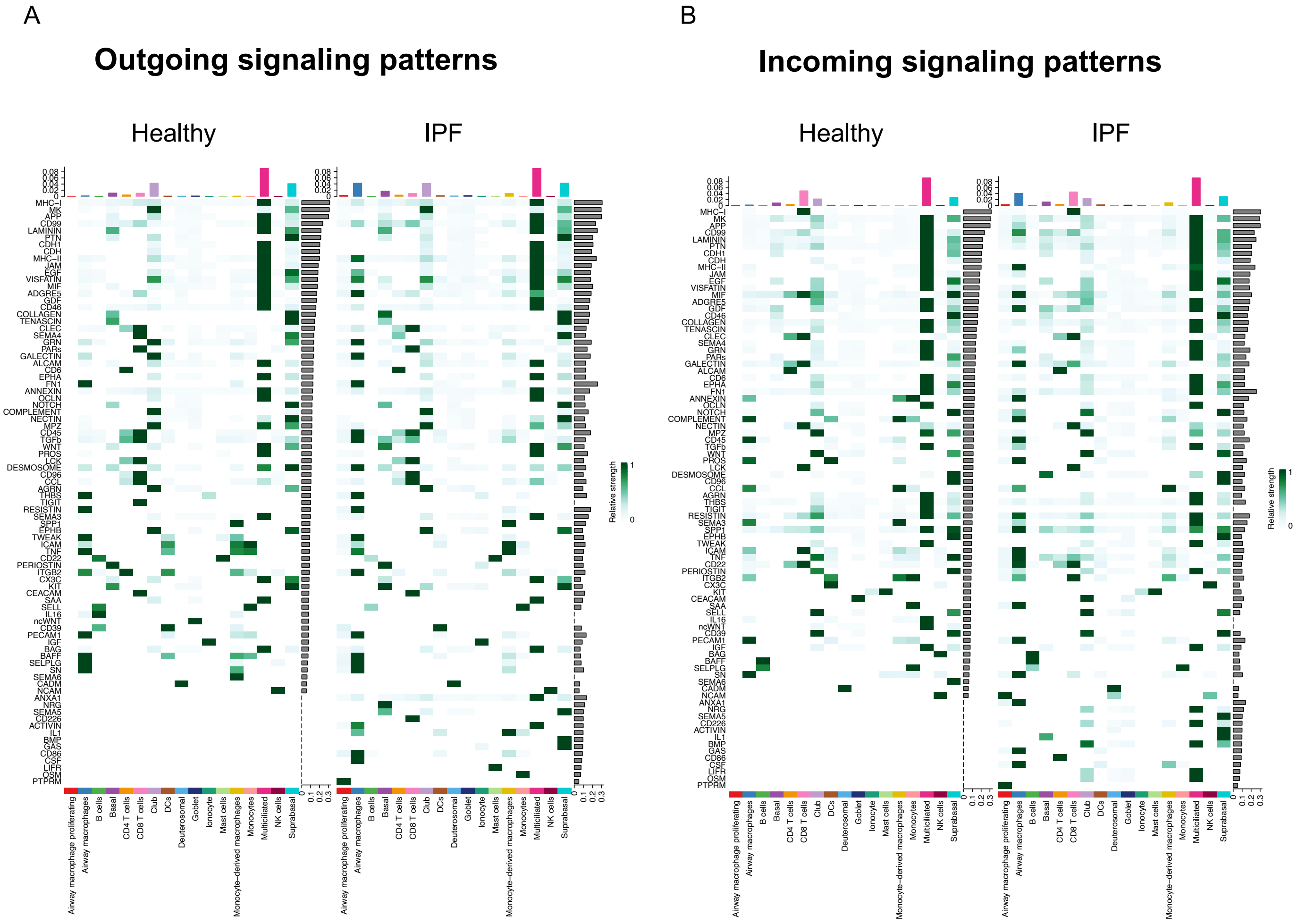

Supplementary Figure 9. Contribution of signalling pathways to outgoing or incoming signalling of each cell population. Heatmaps depict (A) outgoing signalling patterns in healthy controls and IPF patients, and (B) incoming signaling patterns. The heatmaps are coloured based on the relative signaling strength of a signaling pathway across cell groups The values are row-scaled. The top coloured bar plot summarises all signaling pathways displayed in the heatmap by showing the total signaling strength of each cell group. The right grey bar plot shows the total signaling strength of a signaling pathway across all cell groups.

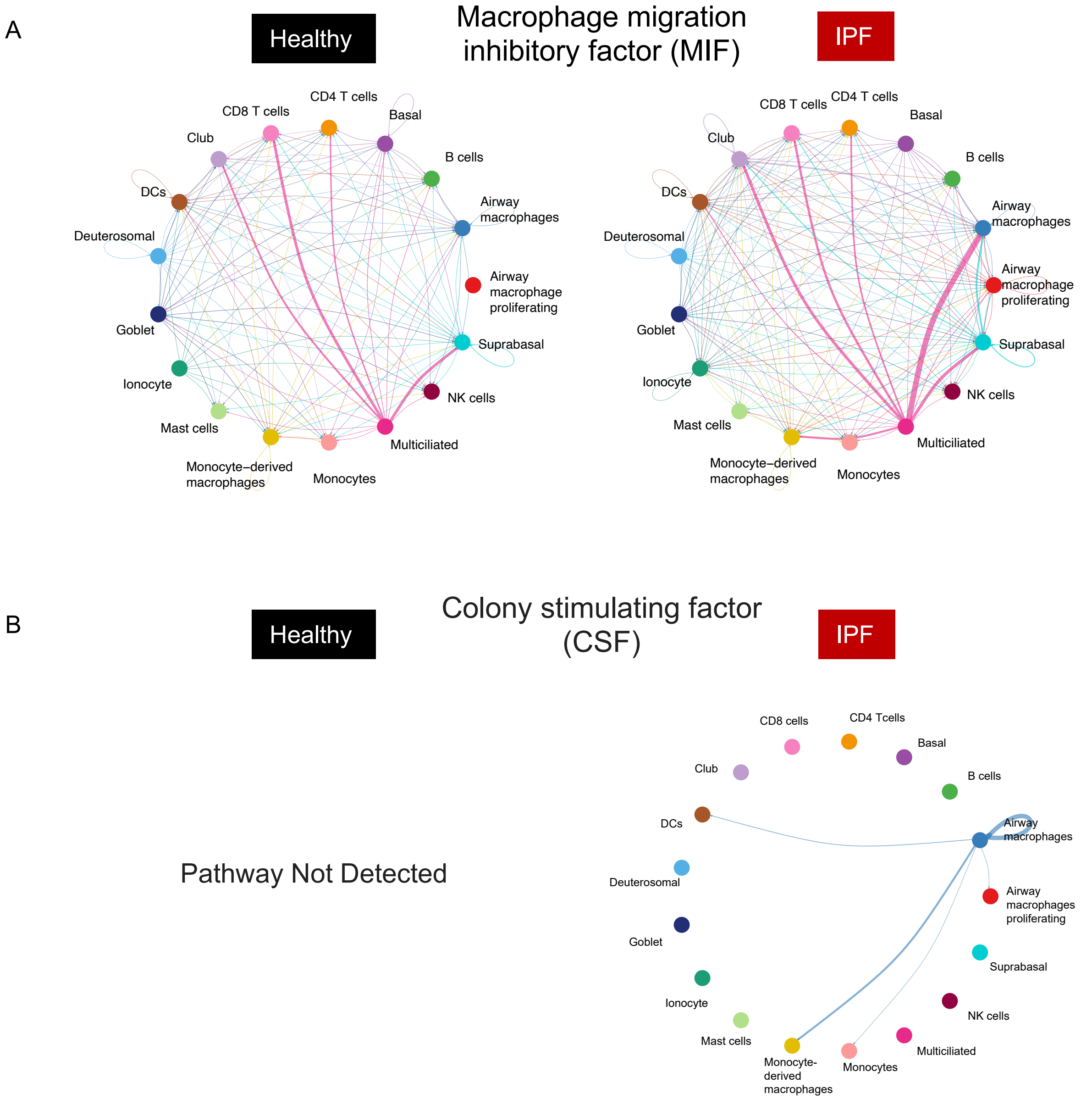

Supplementary Figure 10. Signaling pathway networks potentially driving airway macrophage proliferation in the airway mucosa. Circle plots showing (A) macrophage migration inhibitory factor (MIF) signaling network and (B) Colony stimulating factor (CSF) signaling network in IPF and healthy controls. The weight of edges represents the difference in interaction strength. The colour represents the cellular source of the signalling.

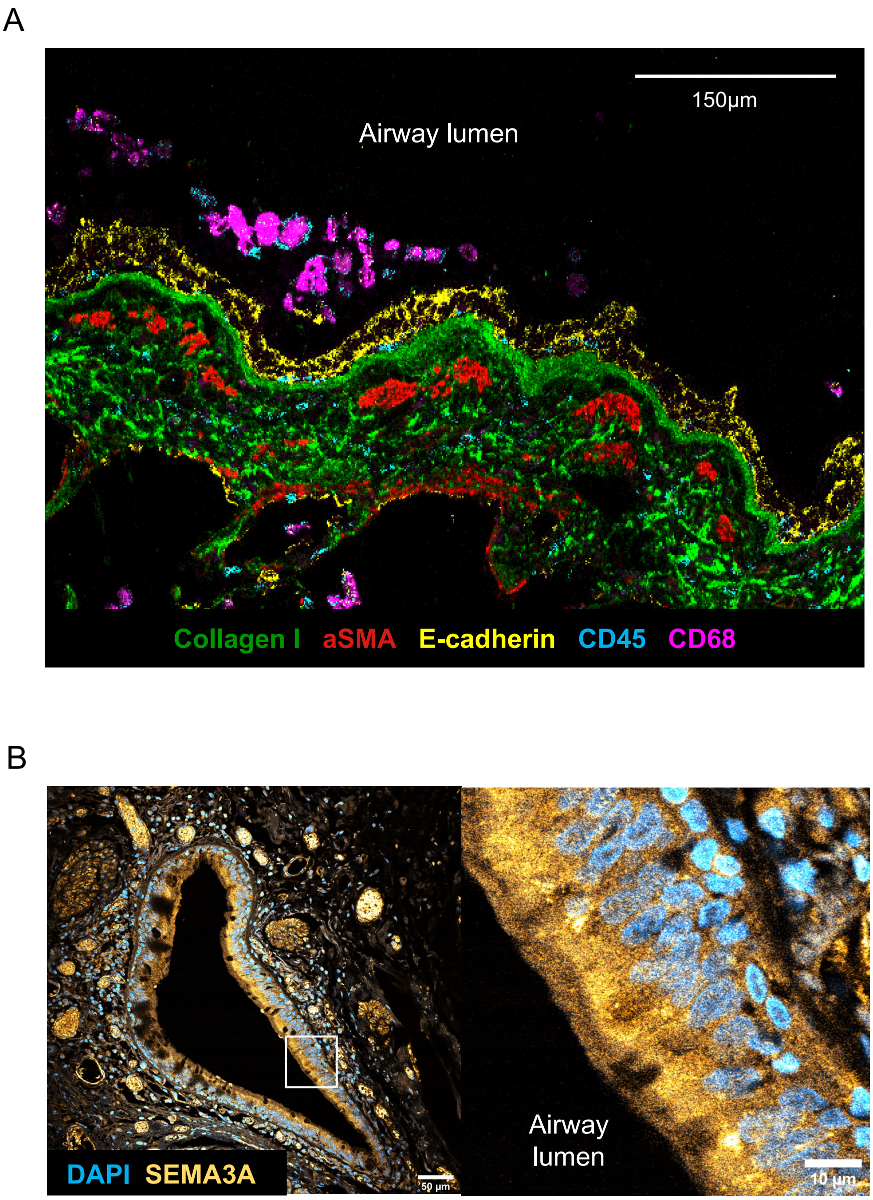

Supplementary Figure 11. Airway macrophages are closely associated with multiciliated cells in the airway lumen. (A) Imaging mass cytometry of control human airway demonstrating airway wall (collagen I, green), stromal cells (alpha SMA, red), multiciliated epithelial cells (E-cadherin, yellow), immune cells (CD45, cyan) and airway macrophages (CD68, magenta). Scale bar, 150µm. (B) Immunofluorescence microscopy of control human lung tissue showing SEMA3A staining in multiciliated cells (left panel; scale bar, 50µm); white box indicates area of high-power zoom displayed in right panel; scale bar, 10µm.

Supplementary Data Tables:

Supplementary Table 1 – Subject-level demographic and clinical data

Supplementary Table 2 – Outcome data for subjects with IPF

Supplementary Table 3 – Cell-type abundance by condition (tab 1), smoking status (tab 2), progression status (tab 3) and survival status (tab 4) using edgeR package

Supplementary Table 4 – miloR cell type abundance analysis

Supplementary Table 5 – Selected differentially expressed genes (DEGs) displayed in Figure 5B using pseudobulk method and Seurat 'FindMarkers' method

Supplementary Table 6 - Differentially expressed genes (DEGs) per cluster in IPF progressors versus IPF non-progressors using glmGamPoi tool

Supplementary Table 7 – Differentially expressed genes (DEGs) per cluster in IPF ex-smokers versus IPF never smokers using glmGamPoi tool
